# Qualitative exploration of the acceptability of a 12-week intervention to reduce sedentary behaviour among ethnically diverse older adults

**DOI:** 10.1136/bmjopen-2024-090384

**Published:** 2025-05-19

**Authors:** Naureen Akber Ali Meghani, Joanne Hudson, Gareth Straton, Jane Mullins

**Affiliations:** 1 Swansea University Faculty of Science and Engineering, Swansea, UK

**Keywords:** Health, Aged, Primary Prevention

## Abstract

**Abstract:**

**Objective:**

The population of ethnically diverse older adults (OAs) is increasing in the UK; this group faces complex health challenges that are exacerbated by language difficulties, socioeconomic status and acculturation experiences. Moreover, this diverse group is the least active and sedentary subgroup within the wider population, which raises a major concern for their health and highlights the need for effective behaviour change interventions to motivate this group to be less sedentary. Therefore, this study aims to explore the acceptability of a 12-week intervention to reduce sedentary behaviour (SB) for ethnically diverse sedentary OAs.

**Design:**

The study employed a qualitative approach to assess the acceptability of the 12-week single-arm intervention for reducing SB.

**Setting:**

The study participants were recruited on a rolling basis from January to May 2024. The recruitment process was conducted through social community organisations and local religious groups in Swansea that provided leisure, sports and recreational activities for ethnically diverse OAs.

**Participants:**

The target population for this study was ethnically diverse OAs aged ≥65 years (including women and men) among (n=20) OAs using in-depth interviews.

**Intervention:**

The intervention consisted of a 40–60 minute personalised one-to-one in-person health coaching session, a wearable activity tracker to remind participants to take breaks from prolonged sitting time, a pamphlet and weekly reminder messages via a mobile phone.

**Primary outcome:**

To assess the acceptability of the intervention.

**Results:**

Reflexive thematic analysis was performed using a deductive approach by integrating four predetermined MRC framework themes. Four overarching themes were included in our analysis: (1) acceptability, (2) usability, (3) functionality and (4) recruitment and retention. OAs were satisfied with the intervention and found it effective and acceptable. The multicomponent intervention provided users with strategies to achieve the goal of reducing their sitting time and provided them with opportunities to be active and independent. In addition, there were personal (eg, health) and social (eg, family) factors that influenced their decision to participate in the intervention.

**Conclusion:**

The findings of this study support the acceptability of the intervention with an ethnically diverse group of OAs. Initial evidence also suggests that the intervention has the potential to increase activity and minimise sitting time in ethnically diverse OAs and therefore will inform a future effectiveness trial. The inclusion of an ethnically diverse population in this study has helped us to understand the needs and challenges of these groups to identify how to design culturally sensitive interventions that are tailored according to their needs. These insights will be incorporated into the planned effectiveness trial.

**STRENGTHS AND LIMITATIONS OF THIS STUDY**

- The current study included a diverse sample with representation of ethnically diverse groups of ≥65 years old to explore their experiences, therefore the generalisability of the findings to wider populations is enhanced.
- The use of qualitative interviews to explore participants’ acceptability of the intervention enabled more in-depth data from older adults.
- There was no control group in the current study, as this was not deemed relevant to address our specific objectives.
- In addition, our participants were not randomly selected and predominantly were well educated, thus generalisation of the results to lower-educated groups might be limited.

## Introduction

 The population of older adults (OAs) is increasing more than any other age group,^1^ with the number of OAs (≥65 years) predicted to double to 1.5 billion by 2050.^2^ This presents a significant public health concern as OAs spend more time being sedentary than other age groups,^3 4^ which has a detrimental effect on successful ageing.^3^ Current estimates showed that OAs sit for more than 10 hours/day^5^ spending 79% of their waking hours being sedentary.^6^ The majority of this sedentary time is spent engaged in recreational activities within the home space, particularly in OAs who are isolated.^7^

Unfortunately, the condition is further aggravated for OAs from socially disadvantaged backgrounds, who are highly physically inactive.^8^ This burden is intensified in ethnically diverse populations who frequently stem from disadvantaged backgrounds and are mostly inactive due to evident health and social disparities.^9 10^ For example, in the UK, black and minority ethnic (BME) groups suffer from a considerably higher disease burden than non-BME communities^11^ and have lower physical activity (PA) levels.^10 12^ A significant concern is raised by the 2022 report’s emphasis on the low PA participation percentages among ethnic minority communities, which stand at 2%.^13^ Given that there are more minority ethnic OAs in Wales and a noticeable increase in ethnically varied OAs in urban areas, this could have possible implications.^14^ Furthermore, OAs from minority ethnic groups are more difficult to reach, less likely to take part in PA interventions, and more likely to discontinue participation in PA programmes.^15^ Therefore, fostering PA and minimising sedentary behaviour (SB) among OAs—especially those from ethnically diverse communities—should be a prime priority worldwide since it permits active ageing and delays the progression of disability and disease.^16^ Further with the advent of COVID-19 constraints, SB has become more pronounced and PA levels have lessened compared with prepandemic levels.^17^,^21^ Similarly, a scoping review also showed that the COVID-19 pandemic has resulted in low levels of activity among black, Asian and minority ethnic communities. Even many OAs are still inactive and sedentary as a consequence of the long impact of the COVID-19 pandemic.^22^ Therefore, minimising SB should be prioritised when promoting active ageing.^23 24^

SB is clearly distinct from inactivity. Inactivity is referred to as standing or exercising insufficiently for extended periods of time and not meeting PA guidelines.^25^ Guidelines on PA and SB^26^ recommend that OAs engage in 150–300 min of moderate–vigorous physical activity (MVPA) or 75–150 min of vigorous PA/week, with at least 2 days of muscle strengthening activity and at least 3 days of varied multicomponent PA that includes strengthening exercises and functional balance, every week. While SB is characterised as the amount of time spent in lower-energy expenditure, such as lying down, sitting or reclining, with a metabolic equivalent of ≤1.5.^26^ A prospective study conducted in the UK determined dose–response associations between sedentary time and 14 distinct non-communicable diseases.^27^ Evidence has linked high levels of SB with metabolic syndrome, blood glucose level, cholesterol level, waist circumference and obesity among OAs.^28 29^ Studies have shown that prolonged SB is associated with the incidence of cardiovascular disease, cancer and type 2 diabetes as well as all-cause mortality, mortality from cardiovascular disease and cancer.^26 30^ Increased SB can also contribute to muscle weakness, reduced bone density and increased frailty in OAs, with these negative health impacts occurring independent of PA.^31 32^ Additionally, high levels of SB can adversely affect OAs’ emotional and physiological well-being^33 34^ and cognitive function.^35^ Several research studies have also documented negative relationships between a broader constellation of screen-based behaviours and mental health.^36 37^ For instance, participants≥50 years who engaged in computer or TV use for >6 hours/day had a higher likelihood of developing depression as compared with participants who used them for <4 hours/day.^36^ Hence, prolonged sedentary time may have particular detrimental effects for OAs,^37^ therefore reducing sedentary time is crucial for both managing pre-existing conditions,^38 39^ as well as for preventing disease and disability,^40^,^42^ meaning it is crucial to take breaks from SB.^43^ Among other nations, the UK, along with the WHO, has now recommended minimising SB as a part of PA guidelines.^44^,^46^ The recent Canadian 24-Hour Movement Guidelines on SB suggest that older individuals≥65 years should break up extended periods of sitting as often as possible.^47^ This recommendation is based on evidence that a focus on promoting PA does not always lead to minimising sitting time, since an individual may satisfy the recommended PA criteria and yet spend a significant amount of time being sedentary.^48^ In this study, time spent in SB is defined as the cumulative daily sitting time.

The UK government makes significant investments to change OAs’ low PA and high SB trends. Nevertheless, current initiatives do not show potential in raising PA levels and reducing SB among OAs from under-represented groups.^49^ This may be because prior research has not focused solely on activities that take place at home,^50^,^56^ but rather on activities that take place outside the home,^57^ which may pose a number of obstacles for ethnically diverse OAs, such as financial hardships, social obligations, a lack of confidence, language barriers, a lack of resources and religious and cultural restrictions.^49^ However, not much research has examined the physical environment of the home in relation to PA and SB levels of OAs. To address this need, we designed a 12-week intervention, which included a brief health coaching session, a booklet, weekly reminder messages and a wearable activity tracker (WAT), aimed to optimise OAs’ home space to maximise PA and minimise SB. This intervention has been fully described in the protocol paper^58^

An important step in implementing this intervention is to assess the acceptability of the intervention for use with ethnically diverse OAs to reduce SB. According to the UK Medical Research Council (MRC), a crucial component in the design of a health-associated intervention is the feasibility/piloting that provides important information on domains like acceptability, usability, functionality, recruitment and retention. This step is considered critical to design and deliver modifications and to assess the acceptability of the intervention to the study population that may enhance the likelihood of intervention effectiveness.^59 60^ Acceptability is a multifaceted construct that reflects people’s perceptions of how appropriate an intervention is in relation to expected or actual cognitive and emotional reactions.^61^ The most appropriate strategy to decrease SB in OAs must be identified because the requirements and expectations regarding activity may vary between age groups (eg, OAs might have different capabilities and motivations than younger groups).^4^ The element of acceptability helps us to understand the extent to which OAs perceived the intervention and how well it met their needs, such as satisfaction, relevance and perceived usefulness (ie, using the intervention to lessen their SB).^62^ This approach will help us to identify the factors associated with the acceptability of the intervention within this population group and investigate OAs’ experience regarding the intervention.^63^ Further for OAs minimising and/or breaking up SB is probably a more acceptable approach than participating in moderate or vigorous PA.^64 65^ In fact, systematic reviews indicated that OAs consider PA as being inconsistent with ageing^65^ and identify exhaustion, discomfort and pain as major obstacles.^65 66^ Additionally, the negative impact of SB on health seems to be highly independent of MVPA.^31 64 67 68^ Therefore, the healthcare interventions that would focus on SB of OAs would be more acceptable and doable. Additionally, the interventions’ acceptability is also significant and must be taken into consideration for effective long-lasting implementation.^69^ Hence, it is important to assess the acceptability of the intervention to the OAs population using a qualitative approach to offer insight into their experiences, perceptions and emotional responses.^59 60^

Currently, a few studies have been conducted with OAs to determine the acceptability of an SB intervention. These intervention studies have mainly integrated one-to-one consultations, feedback provided about behaviour, goal setting, self-monitoring and action planning.^70^,^76^ Preliminary evidence from these trials has shown high acceptance rates in reducing SB. Moreover, participants expressed satisfaction with the intervention, describing it as fascinating, interesting, motivating and feasible.^70^,^76^ However, these SB intervention studies have focused on individuals aged 50 years and above^77 78^ that might be of limited relevance to OAs (≥65 years).^3 4^ According to recent guidelines, OAs are individuals aged 65 years and over.^79^ As a result, the most effective Behaviour Change Techniques for OAs (≥65 years) might be different from those for adults aged 50–64 years.^77 78^ Alongside this, previous research has not captured data from an ethnically diverse sedentary OA group, as engaging multiethnic OAs in research is a challenge, and undoubtedly, they are underrepresented in this research.^80 81^ Given the high prevalence of chronic diseases among this population, research in this group is extremely important^82 83^ as they would arguably benefit most from SB reduction interventions.^70^,^75^ In addition, it is important to obtain OAs’ perceptions before planning an intervention.^3 65 84^ Before designing an intervention, the first phase of our research investigated perceptions of ethnically diverse OAs regarding their activity and sedentary patterns within the home setting using the socioecological model as a theoretical framework.^85^ Through an in-depth analysis of these factors, we gained important knowledge and built insight into the complexity of PA and SB in this group. These findings provided a suitable underpinning to design and execute a multicomponent person-centred intervention for community-dwelling ethnically diverse OAs. This paper aims to assess the acceptability of this intervention and broaden our understanding of the factors related to behaviour change following use of the intervention by ethnically diverse sedentary OAs (≥65 years).

## Methods

### Study design

The study employed a qualitative approach to assess the acceptability of the 12-week single-arm intervention for reducing SB and enhancing activity among OAs using in-depth interviews (IDIs). The first author (NAAM) performed all IDIs and has also delivered the intervention. Though she has undergone rigorous training and has previous experience of conducting qualitative research interviews, which can help to mitigate the potential bias. The study participants were recruited on a rolling basis from January to May 2024, as shown in the flow chart. The study was based in Swansea, Wales and UK.

### Eligibility criteria

The target population for this study was OAs aged ≥65 years (including women and men), who were sedentary (self-reporting sedentary time≥5 hours/day) and who can speak English. OAs were not included if they had a physical impairment that restricted them from taking part in daily living light intensity activity, if they currently participated in the recommended amount of moderate-to-vigorous PA (> 150 min per week) or if they had taken part in a study promoting PA or reducing SB in the preceding 3 months. OAs with psychological impairments and those who were not able to give informed consent or cooperate with the research staff for the entire study period were also excluded.

### Sampling strategy

**Figure 1:**
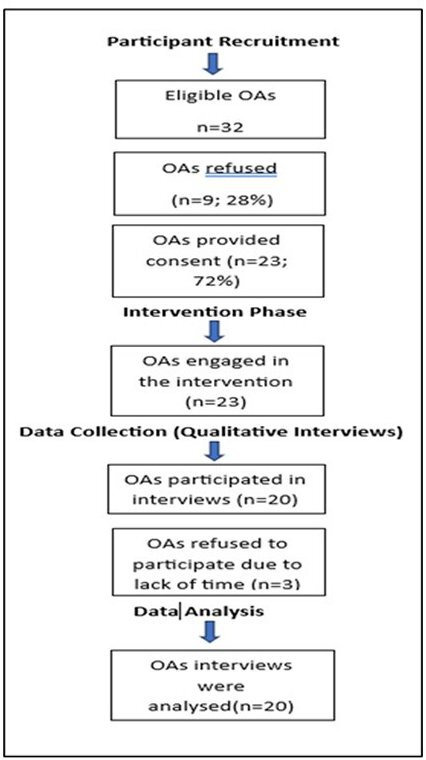
Flow chart of study participants. OAs, older adults.

The study included 23 OAs, of which 20 participants provided qualitative interviews as shown in figure 1. Although this sample size is not determined by statistical power, it was a suitable and useful sample for a feasibility study^86^ taking into account restrictions related to time, money and effort. The sample size used in other feasibility studies of non-pharmacologic therapies that examined comparable results and prompted additional effectiveness trials is comparable to this one.^3 4 75 87^

The recruitment process was conducted through social community organisations and local religious groups in Swansea that provided leisure, sports and recreational activities for ethnically diverse OAs including South-Asians, Africans, Chinese, Koreans and British OAs. Local coordinators and subcoordinators were contacted and acted as the gatekeepers to approach OAs connected to play, sport and community networks, either currently or in the past. The primary author sought permission from the coordinators to inform OAs about the study, and they were asked to communicate their interest to the coordinator in their region. Then the primary researcher contacted the OAs. Information sheets were given to the participants who expressed interest, and the primary researcher discussed the study procedure with them and obtained written consent before conducting the interviews. Figure 1 shows the flow diagram of study participants.

### Patient and public involvement

Patients or the public were not involved in the design, or conduct, or reporting, or dissemination plans of our research.

### Study intervention

The intervention was designed on the basis of the habit formation model.^88^ The concepts of habit formation suggest that decisions to sit are mostly influenced by automatic and unconscious processes.^89 90^ The decision to stand instead of sitting will become further automated with continuous practice, provided that it is initially taken into conscious awareness.^88^ OAs completed measures of PA, SB self-efficacy, self-reporting habits and mental health before and after the intervention. Their sociodemographic characteristics were noted at baseline. The complete details of the intervention can be found in the protocol paper.^58^ Briefly during the 12-week intervention, OAs received a 40–60 min personalised one-to-one in-person health coaching session by the primary researcher, along with a WAT, pamphlet and weekly reminder messages via cell phone. The primary investigator was available via telephone call, messages or email for any questions or issues encountered by the OA participants during the entire study period. The WAT worn on a cord around the neck emitted voice alert messages (eg, “The less I sit, the less stiff I get”; “Keep calm and move within the home” and “Tune your body into fitness”) and vibrations after OAs had been sedentary for 60 min which served as a cue or prompt to avoid extended SB. The goal was to combat SB by using target behaviours: switching extended sitting time with standing or stepping/light activity within their home space to reduce sedentary time. The proposed multicomponent solutions were centred on alerting older individuals to take regular breaks from sitting, which enabled them to develop strategies to change their SB throughout the day.

The individualised health coaching session was conducted to increase OAs’ awareness of physical inactivity and SB and their adverse impacts on the risk of developing chronic disease. Moreover, it was emphasised that breaking up prolonged sedentary time and increasing activity is essential during the entire day. The intervention pamphlet defined SB and PA, their beneficial effects on health and the recommended guidelines for PA and SB. Tips on using the home space to maximise activity and reduce SB were provided. This comprised an everyday cue (eg, “when TV ads…”) and a behaviour to perform in response to the cue (eg, “…stand up or move around within your home”).^70^ To increase the possibility of enactment, these examples offered more or lesser variations of the recommended behaviours or activities to complete at home. The intervention material is attached as online supplemental file 1: intervention material.

### Data collection procedures

On completion of the 12-week intervention, in-depth semistructured exit interviews were conducted by the first author (NAAM; female PhD student) to ascertain the acceptability of the intervention either in person or over the telephone or WhatsApp depending on the preference of study participants. The interview discussed OAs’ perceptions of the intervention, its effect on their behaviour (either activity or SB), any challenges faced and their suggestions for improving the intervention. OAs were asked about all the specific components of the interventions (eg, WATs, health coaching session, pamphlet and reminder messages), and comfortability in using other technologies such as smart phones instead of WATs. Interviews were conducted using the interview guide (online supplemental file 2: interview guide) and lasted 20–40 min. Each interview was audio recorded with the consent of the participant, and the researcher also noted key points.

### Data analysis

Initially, the researcher transcribed all recordings, and interviews taken in Urdu (n=8) were translated into English. Reflexive thematic analysis outlined by Braun and Clarke in 2022 was employed to identify themes and patterns in the data.^91^ Through the deductive approach, four predetermined MRC framework themes that focused on the study’s aims were employed. The key themes include acceptability, usability, functioning, recruitment and retention—are highlighted in the UK MRC framework as being crucial for determining feasibility and piloting health interventions, as explained in the Introduction. Accordingly, the four themes used in our data analysis were chosen especially to support the study’s main aim of determining the acceptability of the 12-week intervention. Familiarisation was the initial step in the process that involved reading and rereading the transcripts and highlighting significant data, such as information that was recurrent across different interviews that was linked with prior research and helped to address the current research aim. Codes were applied to reflect the content of the data, and these were then grouped into subthemes and themes that shared similar meaning. The transcripts were revisited by the researchers to consider and confirm the final names of the themes. In order to ensure transferability, confirmability, reliability and credibility, the researchers carried out the following important steps.^92^ A detailed description of the study context, participant demographics and intervention characteristics was provided. This allows readers to assess the transferability of the findings to other settings or populations. To ensure the reliability of our findings, we employed a rigorous data collection process, including detailed interview protocols and regular check-ins during data collection. Additionally, the same researchers conducted all interviews to minimise variation. Additionally, continuous listening to the interview recordings allowed the researchers to immerse themselves in the topic and obtain an in-depth insight into the discussed themes to ensure confirmability. To assure credibility, we chose transcripts at random for cross-checking during analysis. Selected transcripts were examined by several researchers to confirm topic coherence and consensus in data interpretation. This procedure strengthened our findings’ dependability and credibility and enhanced the analysis quality by taking part in an iterative process.

## Results

20 OAs completed qualitative interviews out of the 23 OAs who were recruited for the 12-week intervention. Three participants refused to provide a formal interview due to lack of time to attend. The mean age of participants was 74±6.4 years and the majority (55%) of the participants were women. A high percentage (65%) of the OAs were retired, with 30% holding a bachelor’s degree. The 2019 Welsh Index of Multiple Deprivation (WIMD) scores, derived from postcodes, were used as a measure of socioeconomic status (SES) due to the unavailability of income and SES data. The WIMD rankings are based on eight deprivation domains: environment, health, employment, housing, income, community safety, education and access to services.^93^ The scores that are obtained range from 1 to 1909, where 1 represents the greatest deprivation and 1909 the least. Participants were split into SES tertiles according to their WIMD Score: low SES (1–636), medium SES (637–1272) and high SES (1273–1909) groups, as it has been previously employed.^94^ In our study, 30% of the older individuals were classified as low SES and medium SES and 40% as high SES based on their WIMD scores. The remaining characteristics of the OAs’ sociodemographic profile are provided in table 1.

**Table 1:** Characteristics of study participants (n=20)

Age	74±6.4
Total sedentary time	543.3±100.1
Gender	
Men	9 (45%)
Women	11 (55%)
Education level	
Completed secondary high school (year 11)	3 (15%)
Trade qualifications/apprenticeship	8 (40%)
Diploma/certificate	3 (15%)
University bachelor’s degree or higher	6 (30%)
Occupation level	
Retired	13 (65%)
Self-employed	7 (35%)
Ethnicity	
Pakistani	5 (25%)
Bangladeshi	4 (20%)
Indian	3 (15%)
African	3 (15%)
British	2 (10%)
Korean	2 (10%)
Chinese	1 (5%)
Family situation	
Single parent household	2 (10%)
Two parent household	18 (90%)
Children living in home	
Yes	9 (45%)
No	11 (55%)
SES strata	
Low	6 (30%)
Medium	6 (30%)
High	8 (40%)
Duration of living in house	
≤5 years	4 (20%)
6–10 years	8 (40%)
>10 years	8 (40%)

SES, socioeconomic status.

Using the deductive approach, four preset MRC framework themes were included: (1) acceptability, (2) usability, (3) functionality and (4) recruitment and retention, shown visually with illustrative quotations in table 2.

**Table 2:** Themes, subthemes and quotations of study participants

Themes	Sub-themes	Quotations
1. Acceptability	Engagement	I must say the intervention was good, I am satisfied with the intervention because …it was meeting my needs. (05F, Indian, Low SES).
	Practicality of the intervention	…I was thinking that you would have told me harder things to do like exercise every day for 20–30 min. It was easier and that was more manageable for people who are at home. (16F, British, Low SES).
	Awareness	My perception has changed a lot…I was unaware of how it can cause harmful effects on different parts of your body… it has made me alert and conscious to avoid being sedentary. (20F, Bangladeshi, Low SES).
	Future reach	…this was my first experience, and I must say it was a good experience even in the future if such interventions take place, I want to be part of these interventions. (07M, Bangladeshi, Low SES).
	Integration with home space	I didn’t make any significant changes or move the equipment within the home. (17F, Pakistani, High SES).
2. Usability	Operationality of multicomponent featuresAccessibility of health coaching session	The first and most important thing in the intervention was teaching which was very helpful because everything that was told to me was helpful…there were many things that I was not aware of… (09F, Pakistani, High SES).
	Appealing features of the pamphlet	I really like the pamphlet. It was well-designed… I pasted it on the front side of my refrigerator … (01F, Korean, Medium SES)
	Feasible reminders; reminder messages	No, it had never annoyed me. Perhaps I had a positive response because it alerts me. When you are doing something …you get engrossed in it. However, the alert acts as a reminder to make me active and I realize that you have to do something for yourself. For example, I stand and drink a glass of water or take a round from my room to the kitchen. Therefore, it was very positive for me. (07M, Bangladeshi, Low SES)
	Technical limitationsStructural features of the WAT	The size of the device is big which makes it heavy, and you have to remove it while you are taking a shower and sometimes you forget to wear it back that is the problem. (10F, Bangladeshi, Medium SES).
	Modification of the WAT	I think to make it more portable…something that is more subtle. Also, for reminders may be the volume could be adjustable. (04F, British, High SES)
	Alternative to WAT	Yes, it is a very good idea that devices can be replaced with smartphones which results in more effectiveness in terms of decreasing sitting time. It will be easier because we can take our phones anywhere and anytime. (18F, Chinese, Low SES).
3. Functionality	Generated outcome: behaviour change	…due to my age, I have stopped doing many activities such as gardening cleaning, cycling, and such kinds of activities but due to this intervention, I have started again… (15M, Pakistani, Low SES).
	Positive impact on health	My muscle strength and my overall functioning have improved, and I am very happy about it. (14F, African, Low SES).
4. Recruitment and retention	Facilitators and barriersLevel of interest	I have noticed that I was very lazy and unable to do much work which encourages me to take part in the intervention … (07M, Bangladeshi, Low SES).
	Role of family	When I discussed this research with my family members, they motivated me to take part as they wanted me to be active. (18F, Chinese, Low SES)
	Role of friends	I think my friends were the source of motivation for me. All of my friends are 75 plus but they are so active. It seems they are in their 40s. This thing encourages me to be active like them and say no to sitting time. (19M, Pakistani, High SES).
	Perception of the research procedures	…I would say the time commitment is a lot that is something you need to keep in mind. If people wanted to take part in this intervention study, they need to be absolutely committed… (08M, Bangladeshi, Medium SES)

SES, socioeconomic status; WAT, wearable activity tracker.

### Theme 1: acceptability

#### Engagement

Participants confirmed the overall acceptability of the intervention. The majority of the OAs verbalised that the experience of engaging in the intervention was quite good as it kept them active. In general, the study participants were satisfied with taking part in the intervention as it was relevant and pertinent to their needs:

I must say the intervention was good, I am satisfied with the intervention because …it was meeting my needs. (05F, Indian, Low SES).

#### Practicality of the Intervention

Engaging in the intervention was considered an enjoyable experience by OAs, which has motivated them to make changes to their lifestyle. They particularly liked that the intervention was based on non-strenuous simple activities that they can perform at their own pace and comfort. In addition, they also found the WAT was helpful in breaking their sedentary time. Therefore, they found the intervention convenient, simple and effective for them:

…I was thinking that you would have told me harder things to do like exercise every day for 20–30 min. It was easier and that was more manageable for people who are at home. (16F*,* British, Low SES).

Yes, reminders should be there because as we get older, we have a tendency to forget everything like we forget to take timely medicine or to break our sitting time. So, in this case, reminders are very useful … (11M, Pakistani, Low SES).

#### Awareness

They suggested that their engagement resulted in a change in their awareness of SB as they became more conscious of the effects of their sitting time and of how frequently they were sedentary:

My perception has changed a lot…I was unaware of how it can cause harmful effects on different parts of your body*…* it has made me alert and conscious to avoid being sedentary. (20F, Bangladeshi, Low SES)

Overall, my experience was very good. I know my sitting time is more, but I never bring this into consideration, and I don’t know how to reduce it. So, your program has given me a wake-up call… (02F, Indian, Low SES).

In addition, OAs shared that accountability was crucial in helping them to break their sedentary pattern. They shared that being part of the intervention made them conscious of breaking their sitting time as it would ultimately be of benefit to them.

… you are part of the intervention then you become a bit more conscious and active. (08M, Bangladeshi, Medium SES)

Some participants reported that breaking sedentary time has automatically become part of their habit change as verbalised by an OA.

…somehow, I have become habitual of it, and I will continue this habit i.e. standing up more. (02F, Indian, Low SES)

#### Future reach

Given the benefits they had experienced, they mentioned continuing even after the intervention has concluded to help them be less sedentary and more active.

Whatever activity I was doing during the intervention, I will continue these activities like stand-up on TV ads, stand-up to cook food…I will continue to do it and make part of my routine activity. (06M, Indian, High SES).

Further, they added that there is potential for longer-term participation in the intervention as shared by the participant.

…this was my first experience, and I must say it was a good experience even in the future if such interventions take place, I want to be part of these interventions. (07M, Bangladeshi, Low SES).

Additionally, culturally adapted, home-based interventions also influence family dynamics and impact healthy behaviour. OAs also noted positive effects of the intervention on their family and suggested that they would recommend the intervention to other OAs:

My family also shows a positive attitude *…* For example, when I stand and walk on reminders they also stand or walk with me. (06M, Indian, High SES).

Certainly, I would recommend this intervention to older adults because I realised that there is a lot that we can do …and bring positive change to our health. (01F, Korean, Medium SES).

#### Integration with the home space

Integrating the intervention into the household was a suitable and culturally sensitive strategy, especially for ethnically diverse OAs. OAs commented on using their home space to engage actively in the intervention, which they found novel and viewed as safe, easy and timesaving and culturally appropriate as they could perform within their home boundary.

The good thing about the intervention is that we have to practice this intervention within our home. We don’t have to go anywhere, we are in a safe zone, we don’t need to spend any money and we don’t need to dress up. We just need to follow the researcher’s instructions and guidance to reduce our sitting time and try to walk more. So, I really like this intervention. (02F, Indian, Low SES).

In addition, engagement in the intervention also promotes optimisation of the home space by the OAs to enhance their daily activity. They added that they have made minor adjustments in terms of interacting with their home environment (ie, moving useless furniture, keeping the WAT device in an accessible place, removing clutter or using stairs).

I have tried to remove clutter from my room so that I can have space in the room, and I can walk easily. (14F, African, Low SES)

However, a few were unable to make any changes within the home space.

I didn’t make any significant changes or move the equipment within the home. (17F, Pakistani, High SES)

### Theme 2: usability

#### Operationality of multicomponent features

##### Accessibility of health coaching session

The multicomponent intervention provided users with strategies to achieve the goal of reducing their sitting time. OAs were positive about the health coaching session as it was informative and helpful in terms of applying different examples to their daily life.

The first and most important thing in the intervention was teaching which was very helpful because everything that was told to me was helpful…there were many things that I was not aware of… (09F, Pakistani, High SES).

…I got to know about guidelines related to physical activity and sedentary behaviour which is kind of a new thing for me. (19M, Pakistani, High SES).

They welcomed the fact that the content was kept simple, easy and without medical jargon to assist them in grasping all the information.

It was very informative*…* If it was longer it would feel like a lecture, so I thought it was good enough. The writing was not too much for me, I think it was fine. I found the perfect content. (16F, British, High SES).

However, a few participants suggested an increase in the number of sessions.

If there are more sessions so it will be more good because we can get more knowledge. (02F, Indian, Low SES)

More sessions because there was only one session in the beginning. Session should be divided throughout the study period. (11M, Pakistani, Low SES)

##### Appealing features of the pamphlet

OAs found the pamphlets beneficial in signposting them to break their sitting time within their home space with the strategies provided in the pamphlet. Moreover, the colourful layout was really appealing and interesting for them.

…the pamphlet was very nice I really like it. it was colourful, the content was written very nicely, and it was more pictorial not that much wordy. The last part where you showed different strategies…I used all these strategies in my daily routine. I liked every part, but the pictorial part was catchy and interesting. (20F, Bangladeshi, Low SES).

I really like the pamphlet. It was well-designed… I pasted it on the front side of my refrigerator … (01F, Korean, Medium SES)

However, some OAs had different opinions regarding the pamphlet:

One is pamphlet, I think it was ok but not very helpful because I referred it in the beginning. (12M, African, Medium SES)

The pamphlet is in paper format; it would be preferable if you make it in the form of a book or chart to secure it and also it would be easy to carry. (19M, Pakistani, High SES)

##### Feasible reminders; reminder messages

One of the intervention components, that is, mobile-phone reminder messages also provided them with alerts to be active, as they noted in their responses:

One thing I found really helpful is the reminder messages*…* I must say they were very helpful…that I need to be involved and engaged in activity. (08M, Bangladeshi, Medium SES).

In addition, some OAs appreciated specific features of the WATs, that is, the vibrations and sound messages that acted as a cue to break their prolonged sitting time. Therefore, they considered these reminders to act as a positive stimulus to be active.

No, it had never annoyed me. Perhaps I had a positive response because it alerts me. When you are doing something …you get engrossed in it. However, the alert acts as a reminder to make me active and I realize that you have to do something for yourself. For example, I stand and drink a glass of water or take a round from my room to the kitchen. Therefore, it was very positive for me. (07M, Bangladeshi, Low SES)

However, it was apparent that in conditions where technology-based approaches are used, ongoing support is imperative. Further discussion in participants’ own languages played a culturally significant role in terms of support and understanding the intervention.

I was in contact with the researcher, she was available 24/7. It was very helpful to discuss in my native language …on a few occasions, I had a few questions, so I directly texted you and called you. Then you just said do it in this way or that way… that was the best part of the whole experiment because continuous help and support were there. (12M, African, Medium SES)

### Technical limitations

#### Structural features of the WAT

On the contrary, the limitations of technological elements were also noted by the users. The size and weight of the WAT made it difficult for the OAs to wear the device continuously. In addition, they needed to remove the WAT for water-based activities, resulting in them often forgetting to put it back on.

The size of the device is big which makes it heavy, and you have to remove it while you are taking a shower and sometimes you forget to wear it back that is the problem. (10F, Bangladeshi, Medium SES).

Some OAs also raised concerns related to the WAT’s vibration and sound messages. They found long vibrations and loud sound messages irritating. Therefore, they often avoid wearing the WAT all the time.

… It’s quite difficult to take around in all the circumstances of life and all daily life. (04F, British, High SES)

In addition, for some, the sound messages generated by the WAT were hard to understand, as shared by a participant.

… and the message was unclear, and I didn’t understand it… (03F, Korean, Medium SES)

#### Modifications of the WATs

OAs who disliked using the WAT were not against the idea of employing these devices in reminding them to lessen their sedentary time and enhance activity within the home. However, they proposed a few suggestions to maximise WAT feasibility. In terms of the device features, OAs suggested developing a lightweight WAT with adjustable sound features or with shorter vibrations for the reminders.

I think to make it more portable…something that is more subtle. Also, for reminders maybe the volume could be adjustable. (04F, British, High SES)

OAs also recommended some changes in the frequency of the reminder timings to receive more reminders to remain active.

I think I would change it in a way that initially it should have the option to give reminders hourly then half an hour then 15 minutes so I could receive more reminders. I think it would have been good for me that I can be more active. (09F, Pakistani, High SES)

However, participants also wanted to add some feedback features in the WAT, along with the reminder, as shared by one of the OAs:

…if there is something that would tell you that you were active for 20 to 30 min in the last 2 hours. So, then you just think oh my God, I was supposed to be active after every 30 minutes, but I am not …so it just gives you some kind of reminders and feedback. Currently, there is no feedback so one can ignore reminders*…* (08M, Bangladeshi, Medium SES)

#### Alternative to WATs

In addition, OAs were asked about comfort and alternatives to using the current WAT as an intervention. Some considered that using a smartphone as an intervention component would be a more feasible and effective approach than the current WAT, and it would keep them more informed about and aware of their activity pattern.

Yes, it is a very good idea that devices can be replaced with smartphones which results in more effectiveness in terms of decreasing sitting time. It will be easier because we can take our phones anywhere and anytime. (18F, Chinese, Low SES).

The continuous wearing of the WAT was also challenging for OAs. Therefore, they recommended designing a non-wearable device.

I think there should be a device that you can keep in your pocket, and it gives you vibrations after every 30 minutes of sitting time. (19M, Pakistani, High SES).

### Theme 3: functionality

#### Generated outcome: behaviour change

OAs in the intervention felt it had a significant beneficial effect on the primary behaviour, that is, minimising SB and increasing PA, hence noting it as an opportunity to be independent:

I think the intervention was helpful in a way because anyhow it has impacted my activity time and sitting time. I tried to move more within my home. I stand up and pick up my stuff, that is, my glasses, shoes, wallet and etc. by myself rather than asking anyone to pick it up for me. (18F, Chinese, Low SES).

They added that it has helped them to identify ways to integrate more activity and engage in active options within their current daily practices, promoting positive changes.

…due to my age, I have stopped doing many activities such as gardening cleaning, cycling, and such kinds of activities but due to this intervention, I have started again… (15M, Pakistani, Low SES).

For some older individuals, they found it hard to overcome their long-time sitting habit as it is a deeply ingrained habit that is linked to their daily life activities including eating, watching TV, talking on the phone, etc. However, they were delighted to make a change in their behaviour by doing more activities:

I hardly stand up and move around the home … it’s a little bit hard for me to shift from sitting behaviour to standing. However, I am happy I am getting rid of sitting time and intervention is working for me. (05F, Indian, Low SES)

#### Positive impact on health

OAs also witnessed a positive impact on their physical health. For instance, they reported their muscle strength, mobility, functioning and energy levels had improved throughout the intervention period. They pointed out that the intervention programme has encouraged them to be more conscious about their health.

My muscle strength and my overall functioning have improved*…* (14F, African, Low SES)

I think intervention study helps me to remember to be more active and take care of my health more. Definitely, it makes me more conscious of my moment and activity level. (04F, British, High SES-04)

In addition, physical breaks from SB improved their mental health and well-being. They reported a reduction in their stress and anxiety levels, leading to an increase in positive feelings and confidence levels.

…when you are working or moving at home… it makes you happy and keeps your mood elevated. (20F, Bangladeshi, Low SES)

You know when you are doing things for yourself you feel good about it. (12M, African, Medium SES)

In terms of habit formation, the duration of the intervention also played a fundamental role, but generally, there were mixed opinions in the present study. The majority of participants believed that the intervention’s duration (12 weeks) was suitable for them. On the other hand, some OAs suggested that the intervention duration should be longer to enable change in their habits. Others suggested that the intervention duration should have been shorter.

I think for real life-changing habits you need to be a bit longer…I was consciously making decisions. But if it had bit longer so it would have become part of my everyday routine. (16F, British, High SES).

I think the duration of the intervention was quite long for me. It needs to be shortened. (17F, Pakistani, High SES)

### Theme 4: recruitment and retention

#### Facilitators and barriers

##### Level of interest

There were personal factors that influenced their decision to participate in the intervention. For example, some OA stated to be healthy and fit was one of the strong motivating factors for them to increase activity and minimise their SB.

I have noticed that I was very lazy and unable to do much work which encourages me to take part in the intervention … (07M, Bangladeshi, Low SES).

In addition, some OAs mentioned pain as a barrier, while some found it an enabler in taking an active part in the intervention:

…I am taking a break from sitting because of that I have noticed that my pain has decreased. (02F, Indian, Low SES)

In the present study, some factors facilitated participation in the intervention study. The provision of adequate and clear instruction was often noted as important to prevent people from dropping out of the study and continuing to engage with the intervention.

No, it was so easy, and yes, I was able to understand those instructions which is why I have brought this much improvements in my life. (10F, Bangladeshi, High SES).

OAs shared the idea of adding incentives to enhance uptake, interest and compensation for their time to willingly participate and enrol in the study, which should take into account in future larger studies:

…I must recommend that there is something for those people who are giving you their time… like appreciating gifts. I mean like give and take situation or like incentive. (12M, African, Medium SES).

### Role of family

The sociocultural factor such as role of family has the possibility to influence the involvement of study participants in the intervention. Through their own set opinions and consequent actions, culturally supported family members may intentionally or unintentionally impact their participation. A few OAs shared that they found their family members were supportive in enrolling and engaging them in the intervention. They added that due to the intervention, they engaged in different daily living activities that satisfied their family members as they avoided being a burden on them.

When I discussed this research with my family members, they motivated me to take part as they wanted me to be active. (18F, Chinese, Low SES)

They were helping and supporting me to take an active part in the intervention program. (13M, African, High SES)

In addition, this study found that their family members were also interested in taking part in the study or had voluntarily participated in the intervention.

They are also motivated to be part of the intervention by seeing me because they want to become active. (10F, Bangladeshi, Medium SES)

So, obviously, my husband understands what the purpose of the study is, so he took part himself. He is very conscious of doing activities. He is taking a more active role than I am taking. (16F, British, High SES).

In contrast, a few OAs highlighted that their family was curious about their recruitment and participation in the intervention, while some shared that their family members got annoyed with the technological component.

Sometimes my family got irritated with the device… (03F, Korean, Medium SES).

Additionally, due to their family responsibilities, there is a lack of engagement in the intervention programme as shared by an OA:

…I get busy with my granddaughter because I look after her, so I often ignore reminders because I am busy with her. (14F, African, Low SES).

### Role of friends

Along with family, friends appear to have a significant impact on OAs’ desire to be active. It was evident from their comments that the OAs are motivated by their friends to enrol and engage in the intervention.

I think my friends were the source of motivation for me. All of my friends are 75 plus but they are so active. It seems they are in their 40s. This thing encourages me to be active like them and say no to sitting time. (19M, Pakistani, High SES).

### Perceptions of the research procedures

Perceptions of the technical component, the time needed to enrol in the study to be more active, and the research procedures were all mentioned as limitations:

…I would say the time commitment is a lot that is something you need to keep in mind. If people wanted to take part in this intervention study, they need to be absolutely committed*…* (08M, Bangladeshi, Medium SES)

Technophobia and some OAs’ ability to use the technology were also questioned:

…they find it very daunting. Even if you say for for example, just putting the watch on the wrist there is nothing else you want to do they find it very challenging because they are scared of technology. (16F, British, High SES).

## Discussion

In this study, we assessed the acceptability of this intervention and broadened our understanding of the factors related to behaviour change following use of the intervention by ethnically diverse sedentary OAs (≥65 years).

Overall, the intervention was highly acceptable among these OAs. They found the multicomponent intervention engaging and satisfying as it was effective and accessible and provided them with insight into their SB. Many OAs appreciated the reminders from the WATs and mobile phone in facilitating them to reduce their sedentary time. Although most participants shared positive experiences with the WAT, they offered suggestions to improve it to make it more age-friendly. The study participants offered insightful input in making the WAT more user-friendly by improving its size and functionality. Their recommendations emphasised the need for a more comfortable fit by making the WAT smaller, improving functionality by including the ability for users to adjust the time period for reminders and the option to modify volume as desired. In order to improve participant engagement, adherence and the overall success of the intervention, it is imperative that these issues be addressed prior to organising an effectiveness trial. These enhancements will help to ensure that the gadget is more appropriate for the requirements of OAs from a variety of ethnic backgrounds. These findings support previous research that OAs will perceive technology as acceptable as long as it is user-friendly.^74^

Awareness regarding SB was one of the most effective ways noted by these OAs to facilitate behaviour change. This study supports previous evidence that information provision may be a promising approach to SB reduction.^4^ Moreover, study participants expressed positive perceptions about the habit reminder strategies that were integrated into daily routines, and many reported the recommended behaviours becoming automatic (ie, habitual) to break up periods of prolonged sitting. This appears to align with predictions from habit theory that, as habit becomes stronger, the habitual behaviour is likely to be more frequently enacted.^95^

In addition, the OA’s environment plays an important role in influencing the process of habit formation. Environment modification can increase habit strength via triggering the cues that are linked to target behaviours in a positive way. It appears beneficial to encourage OAs to create or modify their own personal environment, which will provide them context-specific cues to form healthy habits.^96^ Hence, the possible beneficial effect of modification of the microenvironment can serve as an important step in promoting activity and minimising SB, as highlighted in the current study. Empowering OAs to change their home environments to enhance their contextual circumstances to promote habits of increasing activity and avoiding SB is important,^97^ and the current intervention appears to provide support for OAs to do this. Further integration of these healthy habits has resulted in physical and mental health benefits among OAs in the current study. These findings are consistent with previous qualitative findings^74 98^ that highlighted that doing more activities and minimising SB has given meaning to people’s lives, resulting in a beneficial effect on their health.

Additionally, the home-based implementation intervention ensures that ethnically diverse OAs can participate in it without having to leave their comfortable and familiar surroundings, travel, or spend money. They can get individualised advice without the inconvenience of travel or extra expenses by having healthcare coaching sessions in their homes. Without the pressure of attending outside programmes, pamphlets and reminder messages enable individuals to retain motivation and assimilate material at their own pace, thereby reinforcing healthy behaviours. Self-monitoring is further supported by WAT, which blends in well with daily activities such as housework, prayer or gentle stretching while watching TV. By enabling OAs to maintain their health inside their comfort zone, this strategy not only eliminates logistical and financial obstacles but also honours cultural preferences, making PA more feasible, accessible and long-lasting.

OAs’ recruitment and retention in the intervention were influenced by personal and social factors. Our findings also showed that remaining fit, active and energetic was one of the important motivators of people’s participation in the intervention, supporting previous research that has highlighted that OAs typically cited health-related factors as strong justifications for taking part in an intervention programme.^99^ Additionally, in the present study, we noted different viewpoints on pain related to health conditions that affect their participation, which is in line with previous studies.^100 101^ Hence, in order to improve recruitment and retention in real-life programmes it is important to not only emphasise the health benefits of being active and less sedentary, but also to address personal obstacles such as pain. Reducing SB and maintaining activity levels in older persons can be better supported by real-life programmes that focus on these individual factors.

Alongside the individual factors, sociocultural factors (family and friends) play an important role in engagement with interventions to enhance PA and minimise SB.^102^,^106^ It was made clear in our study that support provided by family members and friends presented a major motivator for enrolment and retention in the intervention for ethnically diverse OAs. However, some familial responsibilities and lack of co-operation were discussed, presenting a potential limitation to OAs’ motivation to participate fully during the intervention period. Incorporating friends and family into the intervention process is crucial for improving recruitment and retention in real-life programmes. Social support networks can be strengthened by employing family-inclusive strategies and peer support groups. Furthermore, removing obstacles can be achieved by flexible scheduling, considering possible family responsibilities and offering carer support resources. Real-life programmes can increase older persons’ long-term adherence by offering a supportive interpersonal setting.

Further, in the present study, providing proper instruction using a culturally adapted native language also helped to minimise misinterpretation and foster a more inclusive and supportive environment, as evident in previous research.^107 108^ Alongside, ongoing support from the researcher facilitated OAs’ continuous engagement and retention with the intervention. Similar strategies can be used in real-world programmes by giving guidance through educational seminars/workshops, keeping ongoing communication to promote engagement and offering support through programme facilitators. In addition, retaining older persons also depends on optimising the benefit-to-burden ratio. This can be achieved in real-world contexts by reducing transportation obstacles by using easily accessible sites or providing transportation assistance, and fostering social engagement opportunities with staff and peers.^81^ A sense of achievement and belonging can also be promoted by acknowledging participants' efforts through modest incentives (eg, postactivity refreshments or vouchers for exercise classes), certificates of participation or recognition through community. Engagement can be further increased by programmes that appeal to altruistic motivations, such as enabling participants to mentor others or improve the well-being of the community. Real-life programmes subsequently have the potential to enhance long-term engagement and retention by addressing both logistical and motivational factors.

Engaging ethnically diverse OAs in activity programmes presents unique challenges^109 110^ that go beyond research settings into real-world implementation.^80 81^ However, the high prevalence of chronic diseases among ethnic minority OAs highlights the significance of developing tailored, accessible and inclusive interventions that target their PA and SB.^82 83^ It is possible that multidiverse populations might have complex and unique experiences and opinions of healthcare and community organisations that influence their participation in intervention programmes. Therefore, special attention needs to be given to social and cultural factors^81^ that include SES, language and the different beliefs which are crucial for creating effective interventions. A more varied OA population in the UK is predicted by current immigration and population trends^111^ therefore it is fundamental to create culturally appropriate programmes that may be modified to meet the needs of various communities. Collaboration with local community leaders, religious institutions and cultural groups is necessary for successful real-life implementation in order to guarantee that programmes are reliable and trusted. Participation barriers can also be decreased by offering language assistance, flexible scheduling and culturally relevant activities. Furthermore, building and sustaining a long-term, inclusive, bidirectional connection with an appropriate community group promotes trust and sustained participation, which eventually results in more successful interventions that can enhance the health outcome of ethnically diverse OA populations.^81^

The present study targeted sedentary ethnically diverse OAs as these individuals may benefit the most from the current intervention. The use of qualitative interviews to explore participants’ acceptability of the intervention enabled more in-depth data to be obtained than would have been generated through questionnaire data, thus giving richer insight into OAs’ perceptions and experiences. Our data offer practical suggestions for SB reduction and highlight potential barriers to the uptake of this intervention that will primarily inform the next stage of our intervention development. With SB and PA interventions, it is clear that no ‘ne-size-fits-all’, particularly in the multidiverse OA population. However, research studies with ageing populations are fairly biased towards including younger adults, with older individuals often being dismissed from ageing research—especially in studies with PA and SB interventions.^112 113^ The current study offers a strength in this regard as we recruited a diverse sample with representation of ethnically diverse groups≥65 years to explore their experiences. Therefore, the generalisability of the findings to wider populations is enhanced. In addition, the team-based, iterative approach to analysing the data improved the rigour of our investigation.

Nevertheless, there are a few limitations to our study. The researcher who delivered the intervention also conducted the post-intervention interviews, thus findings may be influenced by positive demand characteristics, particularly those relating to the delivery of the intervention. However, the researcher has undergone qualitative training and maintained a reflective diary to understand and minimise her biases. In addition, our participants were not randomly selected and predominantly were well educated, thus generalisation of the results to lower-educated groups might be limited. Finally, no conclusions can be drawn about long-term adherence to the intervention as the design did not include long-term follow-up measures and a control group in the current study, as this was not deemed relevant to address our specific objectives. The aim of this study was not to draw conclusions about intervention effectiveness, but rather to use behavioural data as one of several indices of acceptability, on the assumption that positive behaviour change can be taken to indicate that potential participants are willing to respond positively to the intervention. Having OAs involved in the design and delivery of SB reduction programmes (ie, coproduction) appears to be a particularly promising avenue in future studies,^4^ as participants can suggest the range of activities offered to reduce SB.

### Conclusion

The findings of this research that included diverse ethnic OAs have the potential to be implemented to a wider population, promoting health equity and reducing disparities. The inclusion of a diverse ethnic population helps us to understand the needs and challenges of these groups to enable us to design culturally sensitive interventions that are tailored according to their needs. In this regard, the current study makes a substantive contribution to this field by developing an intervention that is home-based and therefore accessible by OAs from different cultural backgrounds and which can accommodate cultural norms that might otherwise influence participation in PA outside the home. The intervention examined here appeared to promote diverse OAs’ activity and is acceptable among sedentary OAs.

Hence, promoting PA and minimising sedentary time among OAs, especially those from ethnically diverse communities, should be a priority worldwide since it allows for active ageing and delays the advancement of impairment and disease.^16^ This intervention can be helpful for community stakeholders and healthcare providers, will raise awareness of PA promotion among organisations and healthcare professionals and can be implemented within their setting to reach a wider audience. While this study has demonstrated the acceptability of the intervention, it is imperative that future studies incorporate the participants’ feedback and test its effectiveness in a larger effectiveness trial.

## Supplementary material

10.1136/bmjopen-2024-090384 online supplemental file 1

10.1136/bmjopen-2024-090384 online supplemental file 2

## Data Availability

Data are available on reasonable request.

## References

[1] Compernolle S, Cardon G, van der Ploeg HP (2020). Engagement, acceptability, usability, and preliminary efficacy of a self-monitoring mobile health intervention to reduce sedentary behavior in belgian older adults: mixed methods study. JMIR Mhealth Uhealth.

[2] World Health Organization (2019). Decade of healthy ageing 2020–2030, Geneva, Switzerland.

[3] McGowan LJ, Powell R, French DP (2021). Older adults’ construal of sedentary behaviour: Implications for reducing sedentary behaviour in older adult populations. J Health Psychol.

[4] Mcgowan L (2020). Developing an intervention to reduce sedentary behaviour in older adults: The University of Manchester (United Kingdom).

[5] Rosenberg D, Walker R, Greenwood-Hickman MA (2020). Device-assessed physical activity and sedentary behavior in a community-based cohort of older adults. BMC Public Health.

[6] Giné-Garriga M, Sansano-Nadal O, Tully MA (2020). Accelerometer-measured sedentary and physical activity time and their correlates in european older adults: the SITLESS study. J Gerontol A Biol Sci Med Sci.

[7] Leask CF, Harvey JA, Skelton DA (2015). Exploring the context of sedentary behaviour in older adults (what, where, why, when and with whom). Eur Rev Aging Phys Act.

[8] Roe J, Aspinall PA, Ward Thompson C (2016). Understanding relationships between health, ethnicity, place and the role of urban green space in deprived urban communities. Int J Environ Res Public Health.

[9] Long J, Hylton K, Spracklen K (2009). Systematic review of the literature on black and minority ethnic communities in sport and physical recreation.

[10] Koshoedo S, Simkhada P, Van. Teijlingen E (2009). Review of barriers to engaging black and minority ethnic groups in physical activity in the United Kingdom. GJHS.

[11] Stevenson J, Rao M (2014). Explaining levels of wellbeing in Black and Minority Ethnic populations in England.

[12] Williams ED, Stamatakis E, Chandola T (2011). Assessment of physical activity levels in South Asians in the UK: findings from the Health Survey for England. J Epidemiol Community Health.

[13] Welsh Institute of Physical Activity, Health and Sport (WIPAHS) (2023). Interim report on the evaluation of the sport wales 60+ active leisure scheme February 2023.

[14] Ethnicity by area and ethnic group. Welsh Government Stats Wales 2023.

[15] O’Regan A, García Bengoechea E, Clifford AM (2020). How to improve recruitment, sustainability and scalability in physical activity programmes for adults aged 50 years and older: A qualitative study of key stakeholder perspectives. PLoS ONE.

[16] Sport England (2022). Active Lives data tables.

[17] Stockwell S, Trott M, Tully M (2021). Changes in physical activity and sedentary behaviours from before to during the COVID-19 pandemic lockdown: a systematic review. *BMJ Open Sport Exerc Med*.

[18] Lefferts EC, Saavedra JM, Song BK (2022). Effect of the COVID-19 pandemic on physical activity and sedentary behavior in older adults. J Clin Med.

[19] Elliott J, Munford L, Ahmed S (2022). The impact of COVID-19 lockdowns on physical activity amongst older adults: evidence from longitudinal data in the UK. BMC Public Health.

[20] Otaki N, Yokoro M, Yano M (2022). Social contact impacts physical activity and sedentary behavior among older adults in Japan due to COVID-19. BMC Geriatr.

[21] Nduka I, Kabasinguzi I, Ali N (2023). The impact of COVID-19 on the changes in health behaviours among Black, Asian and Minority Ethnic (BAME) communities in the United Kingdom (UK): a scoping review. BMC Public Health.

[22] Shanbehzadeh S, Zanjari N, Yassin M (2023). Association between long COVID, functional activity, and health-related quality of life in older adults. BMC Geriatr.

[23] Berthelot G, Johnson S, Noirez P (2019). The age-performance relationship in the general population and strategies to delay age related decline in performance. *Arch Public Health*.

[24] Harvey JA, Chastin SFM, Skelton DA (2018). Breaking sedentary behaviour has the potential to increase/ maintain function in frail older adults. *J Frailty Sarcopenia Falls*.

[25] Panahi S, Tremblay A (2018). Sedentariness and health: is sedentary behavior more than just physical inactivity?. Front Public Health.

[26] World Health Organization (2020). WHO guidelines on physical activity and sedentary behaviour.

[27] Cao Z, Xu C, Zhang P (2022). Associations of sedentary time and physical activity with adverse health conditions: Outcome-wide analyses using isotemporal substitution model. EClinicalMedicine.

[28] Taylor W, Rix K, Gibson A (2020). Sedentary behavior and health outcomes in older adults: A systematic review. AIMS Med Sci.

[29] Saunders TJ, McIsaac T, Douillette K (2020). Sedentary behaviour and health in adults: an overview of systematic reviews. Appl Physiol Nutr Metab.

[30] Ekelund U, Brown WJ, Steene-Johannessen J (2019). Do the associations of sedentary behaviour with cardiovascular disease mortality and cancer mortality differ by physical activity level? A systematic review and harmonised meta-analysis of data from 850 060 participants. Br J Sports Med.

[31] Gianoudis J, Bailey CA, Daly RM (2015). Associations between sedentary behaviour and body composition, muscle function and sarcopenia in community-dwelling older adults. Osteoporos Int.

[32] da Silva VD, Tribess S, Meneguci J (2019). Association between frailty and the combination of physical activity level and sedentary behavior in older adults. BMC Public Health.

[33] González K, Fuentes J, Márquez JL (2017). Physical inactivity, sedentary behavior and chronic diseases. Korean J Fam Med.

[34] Hudgins BL, Hevel DJ, Maher JP (2023). Screen-based and non-screen-based sedentary behaviors are differentially associated with affective states in older adults. Psychol Sport Exerc.

[35] Falck RS, Davis JC, Liu-Ambrose T (2017). What is the association between sedentary behaviour and cognitive function? A systematic review. *Br J Sports Med*.

[36] Madhav KC, Sherchand SP, Sherchan S (2017). Association between screen time and depression among US adults. Prev Med Rep.

[37] Shephard RJ (2011). Physical activity of Canadian adults: Accelerometer results from the 2007 to 2009 Canadian Health Measures Survey. Yearbook of Sports Medicine.

[38] Hajduk AM, Chaudhry SI (2016). Sedentary behavior and cardiovascular risk in older adults: a scoping review. Curr Cardiovasc Risk Rep.

[39] Biswas A, Oh PI, Faulkner GE (2015). Sedentary time and its association with risk for disease incidence, mortality, and hospitalization in adults. Ann Intern Med.

[40] Wilmot EG, Edwardson CL, Achana FA (2012). Sedentary time in adults and the association with diabetes, cardiovascular disease and death: systematic review and meta-analysis. Diabetologia.

[41] Ford ES, Caspersen CJ (2012). Sedentary behaviour and cardiovascular disease: a review of prospective studies. Int J Epidemiol.

[42] Rezende LFM de, Rey-López JP, Matsudo VKR (2014). Sedentary behavior and health outcomes among older adults: a systematic review. BMC Public Health.

[43] Lai T-F, Liao Y, Lin C-Y (2023). Diurnal pattern of breaks in sedentary time and the physical function of older adults. *Arch Public Health*.

[44] Bull FC, Al-Ansari SS, Biddle S (2020). World Health Organization 2020 guidelines on physical activity and sedentary behaviour. Br J Sports Med.

[45] Piercy KL, Troiano RP, Ballard RM (2018). The physical activity guidelines for Americans. JAMA.

[46] Department of Health and Social Care UK chief medical ONicers’ physical activity guidelines.

[47] The Canadian 24-Hour Movement Guidelines (2021). The Canadian 24-Hour Movement Guidelines for Adults aged 65+ Canadian Society for Exercise Physiology Canadian 24-hour movement guidelines: an integration of physical activity, sedentary behaviour, and sleep.

[48] Chastin S, Gardiner PA, Harvey JA (2021). Interventions for reducing sedentary behaviour in community-dwelling older adults. Cochrane Database Syst Rev.

[49] Ige-Elegbede J, Pilkington P, Gray S (2019). Barriers and facilitators of physical activity among adults and older adults from Black and Minority Ethnic groups in the UK: A systematic review of qualitative studies. Prev Med Rep.

[50] Kerr J, Rosenberg D, Frank L (2012). The role of the built environment in healthy aging: Community design, physical activity, and health among older adults. J Plan Lit.

[51] Cunningham GO, Michael YL (2004). Concepts guiding the study of the impact of the built environment on physical activity for older adults: a review of the literature. Am J Health Promot.

[52] Michael YL, Green MK, Farquhar SA (2006). Neighborhood design and active aging. Health Place.

[53] Yen IH, Fandel Flood J, Thompson H (2014). How design of places promotes or inhibits mobility of older adults: realist synthesis of 20 years of research. J Aging Health.

[54] Won J, Lee C, Forjuoh SN (2016). Neighborhood safety factors associated with older adults’ health-related outcomes: A systematic literature review. *Social Science & Medicine*.

[55] Levasseur M, Généreux M, Bruneau J-F (2015). Importance of proximity to resources, social support, transportation and neighborhood security for mobility and social participation in older adults: results from a scoping study. BMC Public Health.

[56] Moran M, Van Cauwenberg J, Hercky-Linnewiel R (2014). Understanding the relationships between the physical environment and physical activity in older adults: a systematic review of qualitative studies. Int J Behav Nutr Phys Act.

[57] Matz CJ, Stieb DM, Davis K (2014). Effects of age, season, gender and urban-rural status on time-activity: CanadianHuman Activity Pattern Survey 2 (CHAPS 2). Int J Environ Res Public Health.

[58] Meghani NAA, Hudson J, Stratton G (2024). A multi-method feasibility trial of a multi-component behaviour change intervention to reduce sedentary behaviour and increase physical activity among ethnically diverse older adults. BMJ Open.

[59] Campbell M, Fitzpatrick R, Haines A (2000). Framework for design and evaluation of complex interventions to improve health. BMJ.

[60] Craig P, Dieppe P, Macintyre S (2008). Developing and evaluating complex interventions: the new Medical Research Council guidance. BMJ.

[61] Sekhon M, Cartwright M, Francis JJ (2017). Acceptability of healthcare interventions: an overview of reviews and development of a theoretical framework. BMC Health Serv Res.

[62] Ayala GX, Elder JP (2011). Qualitative methods to ensure acceptability of behavioral and social interventions to the target population. J Public Health Dent.

[63] Devereux-Fitzgerald A, Powell R, Dewhurst A (2016). The acceptability of physical activity interventions to older adults: A systematic review and meta-synthesis. Soc Sci Med.

[64] Sardinha LB, Ekelund U, dos Santos L (2015). Breaking-up sedentary time is associated with impairment in activities of daily living. Exp Gerontol.

[65] McGowan LJ, Devereux-Fitzgerald A, Powell R (2018). How acceptable do older adults find the concept of being physically active? A systematic review and meta-synthesis. Int Rev Sport Exerc Psychol.

[66] Compernolle S, De Cocker K, Cardon G (2020). Older adults’ perceptions of sedentary behavior: a systematic review and thematic synthesis of qualitative studies. Gerontologist.

[67] Li T, Pan Y, He Q (2023). Associations between sedentary behaviour, physical activity and frailty in older Chinese women: A cross-sectional study. J Clin Nurs.

[68] Sardinha LB, Santos DA, Silva AM (2015). Breaking-up sedentary time is associated with physical function in older adults. J Gerontol A Biol Sci Med Sci.

[69] McCain JE, Caissie L, Edwards J (2023). Long-term care residents’ acceptance of a standing intervention: A qualitative intrinsic case study. Geriatr Nurs (Lond).

[70] White I, Smith L, Aggio D (2017). On your feet to earn your seat: pilot RCT of a theory-based sedentary behaviour reduction intervention for older adults. Pilot Feasibility Stud.

[71] Kerr J, Takemoto M, Bolling K (2016). Two-arm randomized pilot intervention trial to decrease sitting time and increase sit-to-stand transitions in working and non-working older adults. PLoS ONE.

[72] Gardiner PA, Eakin EG, Healy GN (2011). Feasibility of reducing older adults’ sedentary time. Am J Prev Med.

[73] Lewis LK, Rowlands AV, Gardiner PA (2016). Small Steps: Preliminary effectiveness and feasibility of an incremental goal-setting intervention to reduce sitting time in older adults. Maturitas.

[74] Matson TE, Renz AD, Takemoto ML (2018). Acceptability of a sitting reduction intervention for older adults with obesity. BMC Public Health.

[75] Rosenberg DE, Gell NM, Jones SMW (2015). The feasibility of reducing sitting time in overweight and obese older adults. Health Educ Behav.

[76] Matei R, Thuné-Boyle I, Hamer M (2015). Acceptability of a theory-based sedentary behaviour reduction intervention for older adults ('On Your Feet to Earn Your Seat’). BMC Public Health.

[77] French DP, Olander EK, Chisholm A (2014). Which behaviour change techniques are most effective at increasing older adults’ self-efficacy and physical activity behaviour? A systematic review. Ann Behav Med.

[78] French DP, Banafa R, Williams S (2021). How does the understanding, experience, and enactment of self-regulation behaviour change techniques vary with age? a thematic analysis. Appl Psychol Health Well Being.

[79] World Health Organization (2010). Global recommendations on physical activity for health.

[80] McMurdo MET, Roberts H, Parker S (2011). Improving recruitment of older people to research through good practice. Age Ageing.

[81] Mody L, Miller DK, McGloin JM (2008). Recruitment and retention of older adults in aging research: (See editorial comments by Dr. Stephanie Studenski, pp 2351–2352). J Am Geriatr Soc.

[82] Zhou P, Hughes AK, Grady SC (2018). Physical activity and chronic diseases among older people in a mid-size city in China: a longitudinal investigation of bipolar effects. BMC Public Health.

[83] Crnković I, Lončarek K, Železnik D (2023). Relationships between physical activity and selected chronic diseases among functionally independent long-term care residents during the post-lockdown period in Croatia. Int J Environ Res Public Health.

[84] McGowan LJ, Powell R, French DP (2019). How acceptable is reducing sedentary behavior to older adults? Perceptions and experiences across diverse socioeconomic areas. J Aging Phys Act.

[85] Meghani NAA, Hudson J, Stratton G (2023). Older adults’ perspectives on physical activity and sedentary behaviour within their home using socio-ecological model. PLoS ONE.

[86] Whitehead AL, Julious SA, Cooper CL (2016). Estimating the sample size for a pilot randomised trial to minimise the overall trial sample size for the external pilot and main trial for a continuous outcome variable. Stat Methods Med Res.

[87] Fitzsimons CF, Kirk A, Baker G (2013). Using an individualised consultation and activPAL feedback to reduce sedentary time in older Scottish adults: Results of a feasibility and pilot study. Prev Med.

[88] Lally P, Wardle J, Gardner B (2011). Experiences of habit formation: A qualitative study. Psychol Health Med.

[89] Gardner B (2015). A review and analysis of the use of “habit” in understanding, predicting and influencing health-related behaviour. Health Psychol Rev.

[90] Gardner B, Lally P, Wardle J (2012). Making health habitual: the psychology of “habit-formation” and general practice. Br J Gen Pract.

[91] Byrne D (2022). A worked example of Braun and Clarke’s approach to reflexive thematic analysis. Qual Quant.

[92] Campbell K, Orr E, Durepos P (2021). Reflexive thematic analysis for applied qualitative health research. TQR.

[93] Noble M, Wright G, Smith G (2006). Measuring multiple deprivation at the small-area level. Environ Plan A.

[94] Richards AB, Minou M, Sheldrick MP (2022). A socioecological perspective of how physical activity and sedentary behaviour at home changed during the first lockdown of covid-19 restrictions: the HomeSPACE project. Int J Environ Res Public Health.

[95] Rothman AJ, Sheeran P, Wood W (2009). Reflective and automatic processes in the initiation and maintenance of dietary change. Ann Behav Med.

[96] Feil K, Allion S, Weyland S (2021). A systematic review examining the relationship between habit and physical activity behavior in longitudinal studies. Front Psychol.

[97] Wood W, Neal DT (2016). Healthy through habit: interventions for initiating & maintaining health behavior change. Behav Sci Policy.

[98] Greenwood-Hickman MA, Renz A, Rosenberg DE (2016). Motivators and barriers to reducing sedentary behavior among overweight and obese older adults. Gerontologist.

[99] Fleig L, McAllister MM, Chen P (2016). Health behaviour change theory meets falls prevention: Feasibility of a habit-based balance and strength exercise intervention for older adults. Psychol Sport Exerc.

[100] Bjornsdottir G, Arnadottir SA, Halldorsdottir S (2021). Physical activity of older women living in retirement communities: capturing the whole picture through an ecological approach. J Geriatr Phys Ther.

[101] Poveda-López S, Montilla-Herrador J, Gacto-Sánchez M (2022). Wishes and perceptions about exercise programs in exercising institutionalized older adults living in long-term care institutions: A qualitative study. Geriatr Nurs (Lond).

[102] Costello E, Kafchinski M, Vrazel J (2011). Motivators, barriers, and beliefs regarding physical activity in an older adult population. J Geriatr Phys Ther.

[103] Stathi A, Gilbert H, Fox KR (2012). Determinants of neighborhood activity of adults age 70 and over: a mixed-methods study. J Aging Phys Act.

[104] Hardy S, Grogan S (2009). Preventing disability through exercise: investigating older adults’ influences and motivations to engage in physical activity. J Health Psychol.

[105] Marthammuthu T, Hairi FM, Choo WY (2021). A qualitative investigation on the roles of social support on physical activity behaviour among the rural-dwelling older women in Malaysia. Int J Environ Res Public Health.

[106] Morgan GS, Willmott M, Ben-Shlomo Y (2019). A life fulfilled: positively influencing physical activity in older adults - a systematic review and meta-ethnography. BMC Public Health.

[107] Northridge ME, Shedlin M, Schrimshaw EW (2017). Recruitment of racial/ethnic minority older adults through community sites for focus group discussions. BMC Public Health.

[108] Feldman S, Radermacher H, Browning C (2008). Challenges of recruitment and retention of older people from culturally diverse communities in research. Ageing Soc.

[109] Milani SA, Cottler LB, Striley CW (2023). Perceptions of research participation among a sample of Florida residents aged 50 and over reporting dementia. Ageing Int.

[110] Bowling CB, Whitson HE, Johnson TM (2019). The 5Ts: preliminary development of a framework to support inclusion of older adults in research. J Am Geriatr Soc.

[111] U.S. Census Bureau (2000). Population projections of the united states by age, sex, race, hispanic origin, and nativity: 1999 to 2100.

[112] Brach M, Moschny A, Bücker B (2013). Recruiting hard-to-reach subjects for exercise interventions: a multi-centre and multi-stage approach targeting general practitioners and their community-dwelling and mobility-limited patients. Int J Environ Res Public Health.

[113] Brach M, de Bruin ED, Levin O (2023). Evidence-based yet still challenging! Research on physical activity in old age. Eur Rev Aging Phys Act.

