# Supplementary material for: Qualitative exploration of the acceptability of a 12-week intervention to reduce sedentary behaviour among ethnically diverse older adults
Source: BMJ Open. 2025 May 19;15(5):e090384. doi: 10.1136/bmjopen-2024-090384 (PMC12090861; doi:10.1136/bmjopen-2024-090384)
Supplement: online supplemental file 1 [file bmjopen-15-5-s001.pdf]

PHYSICAL ACTIVITY & SEDENTARY BEHAVIOUR WITHIN HOMESPACE

1

Aims

- Discuss physical activity (PA) and its impact on health
- Explain recommendations OF PA
- Discuss sedentary behaviour (SB) and its impact on health
- Explain Canadian 24-Hour Movement Guidelines for Adults
- Mention examples of reducing SB within home space

2

Physical Activity

Regular activity is not like protect your heart. You can use it as a way to catch up with friends or meet new people. It improve your general health and well-being.

All household chores are counted in activity.

Intensity Of Physical Activity

Sedentary: Not moving working at desk

Light activity: Cleaning, carrying out rubbish, yoga

Moderate activity: Walking, cycling and shopping

Vigorous: Playing football, dancing, swimming

Adults aged 18–64 years

*at least 150–300 minutes of moderate-intensity aerobic physical activity;

*or at least 75–150 minutes of vigorous-intensity aerobic physical activity; or an equivalent combination of moderate- and vigorous-intensity activity throughout the week

*should also do muscle-strengthening activities at moderate or greater intensity that involve all major muscle groups on 2 or more days a week, as these provide additional health benefits.

*replacing sedentary time with physical activity of any intensity (including light intensity) provides health benefits.

5

Benefits of PA; Protect from

Dementia	Hip facture	Depression	Cardiovascular disease
Diabetes	Colon cancer	Breast cancer	All-cause mortality

6

Sedentary Behaviour (SB)

Sedentary behaviour includes activities we do in a seated or reclining position and that don't use much energy. For this program, we often refer to sitting but we also mean any activities that are done while reclining or lying down (other than sleep).

7

SB impact on health

High Blood pressure	Increase risk of chest pain and heart attack	Risk of getting diabetes	Weight gain and obesity
Increase risk of cancer	Depression	Inflammation	Dysfunction of Immune system
	Weak Bones and increased risk of fractures	Decrease in muscle mass and strength	

8

Canadian 24-Hour Movement Guidelines for Adults

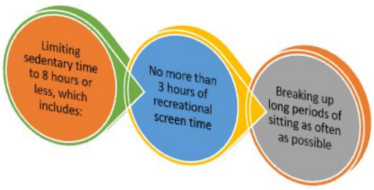

9

Staircase Approach

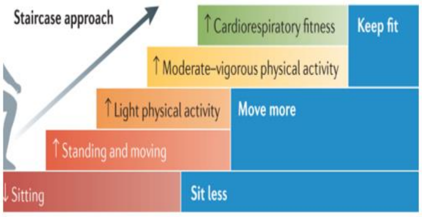

10

Examples

If we are sitting down but using a fair amount of energy, this is **NOT** classed as sedentary behaviour

Sedentary	Not Sedentary
Sitting/lying and watching TV	cycling
Sitting whilst reading or knitting	Chair-based exercises
Driving a car/sitting on public transport	Standing to cook, wash-up or clean
Sitting and using a computer	Any standing! (even when not using a lot of energy)

It is important to note that **sedentary behaviour** is **not** simply **a lack of physical activity**. A person who runs 5km every day but sits for the majority of the rest of the day is both very physically active and very sedentary.

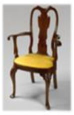

Example 1: many office workers spend a large proportion of the day sitting at a desk, but may go to the gym numerous times a week

Example 2: Parents of toddlers may not have the opportunity to do much physical activity, but also may not get chance to sit down a lot during the day

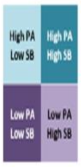

Ways of reducing SB

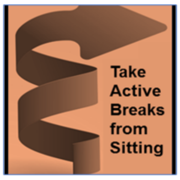

- **Take breaks from sitting every 30 minutes**
- **Stand and move more throughout the day, building towards 30 minutes twice per day.**
- **This help to reduce your sitting time by about 2 hours per day**

13

Taking a Whole Day Approach

Thinking about Reducing Sitting Time: Taking a Whole Day Approach

- One of the first steps is to think about your whole day. In order to sit less and move more, it's important to do some standing and moving activity throughout the whole day
- What times of day do you sit the least and the most? What are you doing during these times? What do you notice (such as feeling tired/awake, in a good/bad mood, etc.)?

Times when I sit the least:	What am I doing?	What do I notic
______ am/pm to ______ am/pm		
Times when I sit the most:		
______ am/pm to ______ am/pm		

14

Make activity: your habit

- Add physical activity into your usual sitting routine and everyday routines. You can develop a new activity habit by doing a specific activity repeatedly at the same point in your routine

Stand, stretch, or move around the room during TV commercials

Set a reminder you to take a break from sitting

Chest and arm stretch, and toe stand when cleaning home

Making Home space conducive for PA

Remove clutter to walk → Place washing liquid on a lower shelf, bend your knees to pick it up → Create space within home for workout

Mow your lawn for PA → Set counter or desk to stand up & cook food → Remove clothes from activity devices & move in accessible place

Making Home space conducive for PA

Stand & wash
- Stand & wash dishes by hands

Gardening
- Start gardening

Get out
- Get out your chair to pray

Set
- Set schedule to avoid screen

Exercise
- Exercise using virtual modality

Stand & wash
- Stand & wash dishes by hands

17

Do certain moves while taking breaks from screen

Using standing desk for different activities

Avoid screen in bedroom

18

With pacing, avoid doing lot of activities

Activity	Gardening all morning	Reset

Instead, when you pace yourself, space your activities out over the day with lots of breaks to rest in between like this

**Get equipment together 15 mins**	**Reset 15 mins**	**Bring equipment outside 10 mins**	**Reset 15 mins**	**Weed for 5-10 mins**	**Reset 15 mins**	**Weed for 5-10 mins**	**Reset 15 mins**

Apply pacing to your activity to sit less and move more

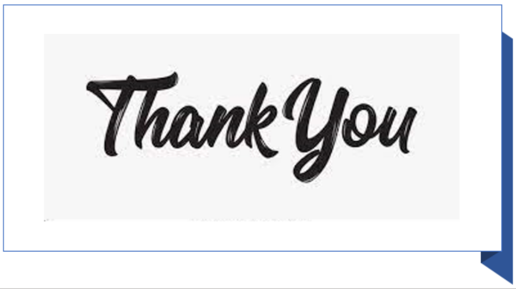

MAKE YOUR MOVE- SIT LESS

1.What counts as activity?

When we talk about being physically active, we don't mean putting on sports kit and joining the gym. You don't have to get hot and sweaty. We really are talking about getting up and moving around more. All these activities will help to improve your health.

2.How much activity?

*at least 150–300 minutes of moderate-intensity aerobic physical activity;

*or at least 75–150 minutes of vigorous-intensity aerobic physical activity; or an equivalent combination of moderate- and vigorous-intensity activity throughout the week

*should also do muscle-strengthening activities at moderate or greater intensity that involve all major muscle groups on 2 or more days a week, as these provide additional health benefits

*replacing sedentary time with physical activity of any intensity (including light intensity) provides health benefits.

3.What type of activity?

Sedentary: Not moving working at desk

Light: Cleaning, yoga, carrying rubbish

Moderate: Walking, cycling, shopping

Vigorous: Playing, dancing, swimming

Very vigorous: Weight exercises, press ups.

- **Protect from:**
- Dementia
- Diabetes
- Colon Cancer
- Breast Cancer
- Hip Facture
- Depression
- Cardiovascular disease
- Cardiovascular mortality
- All-cause mortality

5.What is Sedentary behaviour?

It includes activities we do in a seated or reclining position and that don't use much energy. For this program, we often refer to sitting but we also mean any activities that are done while reclining or lying down (other than sleep).

6. What are the Canadian 24-Hour Movement Guidelines?

*Limiting sedentary time to 8 hours or less, which includes: No more than 3 hours of recreational screen time.

*Breaking up long periods of sitting as often as possible

The best time to make a change is now. Think about what you can do right now to be active. You might not feel like it to start with, but it gets easier over time, and in the end will just be a habit that you do without thinking about it.

7. If we are sitting down and using a fair amount of energy, this is NOT classed as sedentary

Sedentary	Not Sedentary
Sitting/lying and watching TV	Cycling
Sitting while reading or knitting	Chair-based exercises
Driving a car/sitting on public transport	Standing to cook, clean or wash-up
Sitting and using a computer	Standing position

It is important to note that sedentary behaviour is not simply a lack of physical activity. A person who runs 5 km every day but sits for the majority of the rest of the day is both very physically active and very sedentary.

9. Using your Home Space for activity?

Think about when and where you can be physically active. Making some small changes to your daily routine can make a big difference.

- Remove clutter from rooms.
- Intentionally keep stuff on a lower shelf to pick it up.
- Mow your lawn.
- Create space for activity.
- Avoid screens.
- Use stairs within home.
- Remove clothes from exercise device and use it
- Keep exercise device in an accessible space
- Stand & wash dishes
- Set counter and stand up to cook
- Get out of chair to pray.
- Stand up during calls.
- Stand up during selfcare activities.
- Perform exercise moves when boiling kettle.
- Stand up or walk during TV ads.
- Playing with grandchildren
- Spilt up chores/ Pacing (Activity, rest & repeat)

MAKE ACTIVITY PART OF YOUR HABIT

- **Carry on at your own pace**
- **Go slow and start light activity**
- **Note improvement in your activity**
