# Supplementary material for: Qualitative exploration of the acceptability of a 12-week intervention to reduce sedentary behaviour among ethnically diverse older adults
Source: BMJ Open. 2025 May 19;15(5):e090384. doi: 10.1136/bmjopen-2024-090384 (PMC12090861; doi:10.1136/bmjopen-2024-090384)
Supplement: online supplemental file 2 [file bmjopen-15-5-s002.docx]

**In-Depth Interviews**

**Acceptability**

1. What has your experience been with using multi-component intervention (pamphlet, wearable device and health coaching session)?

**Probes:**

a) What do you like the most about this intervention program?

b) What features/ components do you find most useful? – why?

c) What do you like the least about this intervention program?

d) To what degree does the intervention program meet your expectations?

1. Did you ever receive automated reminders to break your sedentary behaviour?

**Probes:**

a) What do you think of these?

b) Do you like the reminders or find them annoying?

c) What modifications do you want in the automated reminders via wearable device ?

**Demand**

1. Tell me about your actual use of intervention program?

a). Has intervention caused you to break sitting time?

b). How often do you consciously do it?

c). Which element of intervention helps you to break sitting time?

 2. What do your family and friends think about you being part of the intervention program?

a) Are they encouraging?

b) Do they see the value it in?

3.What would encourage OA to take part in study?

a) Would you recommend intervention programs to other older adults?

b) If you were asked, would you take part in a larger trial using the intervention? If no, why not? (here you can probe which parts of the intervention would deter them from being part of a larger/longer study).

**Implementation**

    1. What support was available to help you use the intervention program?

a) What is your opinion of the instruction you received?

b) Was it easy to use the activity monitoring devices?

a) Are there any features of the intervention that you find difficult to use?

2. Have you had any problems with the equipment functioning?

a) What did you do to resolve it?

b) Was the problem easily solved?

3. Tell me the factors that positively or negatively influenced intervention use?

**Practicality**

1. Please explain how you use the intervention program within your daily activities.

2. How would you feel if, to use the activity device, you had to use your own personal Smartphone?

a) Do you expect it to be easier or more difficult to use? And why?

b) Would this change the way you use the activity device or incorporate it into your daily activities?

c) Do you think you would have required different instruction and guidance?

d) Would this change your willingness to use the activity device?

3. Could you think of other ways, besides using intervention, that would help you to increase physical activity and minimize sedentary behavior? If yes, what are they?

**Limited Efficacy**

1. How would you find your relationship with research staff?

a) Do you feel that they support you in your activity or care?

2. In what ways the intervention program has changed your behaviour to manage your:

- Physical activity?
- Sedentary behaviour
- Health and well-being

3. In general, what would make the intervention program better?

4. Are there any other important issues or anything you would like to add that we haven’t discussed?

Thank you so much for allowing me to come and interview you. Your time and your views are greatly appreciated. All the information you've provided will be very helpful in shaping future research, policy and programs in the area of older adults’ activity, housing and health.
