# Supplementary material for: Qualitative exploration of the acceptability of a 12-week intervention to reduce sedentary behaviour among ethnically diverse older adults
Source: BMJ Open. 2025 May 19;15(5):e090384. doi: 10.1136/bmjopen-2024-090384 (PMC12090861; doi:10.1136/bmjopen-2024-090384)
Supplement: Supplementary file 3 [file bmjopen-2024-090384.reviewer_comments.pdf]

PEER REVIEW HISTORY

BMJ Open publishes all reviews undertaken for accepted manuscripts. Reviewers are asked to complete a checklist review form and are provided with free text boxes to elaborate on their assessment. These free text comments are reproduced below.

ARTICLE DETAILS

Title (Provisional)

A qualitative exploration of the acceptability of a 12-week intervention to reduce sedentary behaviour among ethnically diverse older adults

Authors

Meghani (NAAM), Naureen Akber Ali; Hudson (JH), Joanne; Straton, Gareth; Mullins, Jane

---

VERSION 1 - REVIEW

**Reviewer**	**1**
**Name**	**Hao, Yufang**
**Affiliation**	**Beijing University of Chinese Medicine, School of Nursing**
**Date**	**18-Aug-2024**
**COI**	**none**

This manuscript is a study of great public health significance, focusing on the improvement of health behavior of multi-ethnic elderly groups. The research design is reasonable and the intervention measures are clear, which can effectively evaluate the behavior changes of the target population. The advantage of the manuscript lies in its concern for multi-ethnic groups, which helps to reveal the influence of cultural factors on the acceptability of intervention. However, the manuscript still needs to be improved in some aspects, especially in the preface, the clear definition of acceptability and sedentary, the detailed description of methods, the specific analysis of results, and the logic and depth of discussion.

1 Title

1) Punctuation should not be added at the end of the title, and it is suggested to delete it.

2) The intervention strategy in this paper is designed to reduce sedentary behavior. In fact, the intervention measures to reduce sedentary behavior may not necessarily increase physical activity (similarly, the intervention measures to increase physical activity may not necessarily reduce sedentary behavior). Therefore, the title mentions reduce sedentary behavior and increase physical activity, which needs to be considered again.

2 Abstract

1) Introduction: It is suggested to emphasize the importance of sedentary behavior and physical activity in multi-ethnic elderly groups, and point out the research gaps in this field to highlight the necessity of research.

2) Methods: It is suggested to adjust the expression of "Underpinned by the themes discussed below", which can be directly defined as "four pre-determined MRC framework themes" to improve the clarity of expression. Also, it is suggested that the number of participants be clearly stated.

3) Conclusion: The conclusion should be consistent with the purpose of the study and emphasize the influence of the study on the multi-ethnic elderly. Moreover, the abbreviation "SB" should be marked with its full name for the first time.

3 Introduction

1) The preface mentions the concepts of sedentary behavior and physical activity, but it lacks a clear distinction. It is suggested to add a paragraph to explain the difference between the two concepts of "sedentary behavior" and "insufficient physical activity" in order to avoid confusion among readers.

2) Please define the specific time of sedentary behavior in this study, which is the cumulative daily time or the single sitting time?

3) Although the author mentioned the importance of "acceptability", he did not clearly define this concept and did not fully elaborate its significance. This is an important aspect that needs to be improved. Please add more explanations and provide relevant quotations.

4) The first paragraph mentioned "There has been an increase in concern about these findings following the imposition of COVID-19 constraints during the pandemic, as sedentary behaviour (SB) has become more pronounced and physical activity (PA) levels have lessened compared to pre pandemic levels (8).", but this study was actually carried out after the epidemic, so it is suggested to supplement the latest data or research on the changes of physical activity level after the epidemic to provide more complete background information.

5) It is suggested that it is necessary to further elaborate the intervention research on sedentary behavior and physical activity in multi-ethnic elderly groups. For example, supplement the data related to sedentary epidemic of ethnic minority elderly people, emphasize how the specific health challenges and cultural differences of these groups affect their health behaviors, and how the current research can fill the gaps in this field.

6) Page 4, lines 18-22, lines 51-53, etc., please provide necessary references.

7) The appearance of MVPA in introduction does not specify what it means.

4 Methods

1) It is suggested that the author supplement the research design flow chart to show the research process intuitively.

2) The ethical approval number is missing in the study design, please add it.

3) Rationality of including the standard: in the part of "self-reported sedentary time ≥5 hours a day", the author needs to explain the basis for choosing this standard (whether it is based on previous research or guidelines) and its relevance to acceptability research.

4) In the section of "Patient and public involvement", please describe the patient and public participation in the whole research process, not just setting the research agenda. If there is no PPI, it is suggested to explain why there is no PPI and how this may affect the research.

5) In the section of "Data Analysis", it is said that "four pre-determined MRC framework these are focused on the study's aims were employed", and it is suggested to clearly explain how to determine these four topics from the MRC framework and explain their relevance to the research objectives.

6) Please supplement the process or standard for determining the sample size. The proposal explains how to determine that data saturation has been achieved, especially considering the different views that may be brought by multi-ethnic participants.

7) Research intervention: Although the author mentioned that intervention is based on habit forming model design, he did not clearly describe how to form model design based on habit, and suggested adding it to increase clarity. At the same time, it is suggested to provide the details of the research intervention in the form of attachments, including the specific use methods, examples and implementation details of each component of the intervention.

5 Results

1) The naming and division of topics and sub-topics need further discussion: a. It is suggested to adjust the naming of sub-topics under "Theme 1: Acceptability" to make them consistent in bias. For example, change "Effective and Simple" to neutral description. B the sub-topic "Convenience" under "theme 1: acceptability" overlaps with some other sub-topics, so it is suggested to re-examine and integrate the content; C. The sub-theme "Habit automation and conscious habit" under theme 3 suggests that "Habit Automation" and "Conscious Habit" should be merged or further condensed to improve the clarity; D for "Mediators" in theme 4, it is suggested to clarify whether its definition and use are appropriate.

2) The results lack the specific results of multi-ethnic groups, so it is suggested to report the special performance of multi-ethnic groups in the results and discuss the influence of cultural factors on the acceptability of intervention.

3) It is suggested to describe the feedback of subjects to different components of multi-component intervention in order to better understand the effectiveness and acceptance of each component.

4) It is suggested that the author explain the quality of qualitative research, how to transfer, reliability, confirmability and credibility.

5) TABLE 1 does not provide the time for study participants to sit every day, please add it.

6 Discussion

1) The third paragraph of the discussion lacks clear argument logic. The paragraph begins to discuss the importance of cognitive improvement, then jumps to habit formation, then to the role of reminding, and finally returns to the duration of intervention. This leap-forward discussion makes the central argument unclear, and it is suggested to reorganize the paragraph structure. In addition, this part mentioned the theory of habit formation, but did not fully explore how this theory specifically explained the research findings. The comparison with the existing literature is also general, lacking specific research citations and in-depth comparison.

2) 2) Similarly, in the third paragraph of the discussion, it is mentioned that reminding helps to automate behavior, but at the same time it is said that continuous reminding is needed. The relationship between these two viewpoints needs to be explained more clearly, because they actually describe different stages of behavior change and habit formation. In addition, it is mentioned that "with the passage of time, these may no longer be needed, because standing has become more automatic and habitual." "This statement is vague and fails to explain how long it will take to form a new habit. It is difficult to set intermediate checkpoints or evaluate progress standards for follow-up studies that lack time indicators.

3) "This is aligned with the previous qualitative findings that identified OAs were interested in using technology as long as it is user-friendly."is mentioned in the first paragraph on page 17, please supplement the relevant references.

4) Consistent with the suggestions made in the results section, it is suggested to discuss the specific results of multi-ethnic groups in depth and analyze the significance and challenges of these characteristics in the intervention, especially how cultural factors affect the acceptability of the intervention.

5) Consistent with the suggestions in the results section, it is suggested to discuss the acceptability of different components of multi-component intervention in detail, and point out which components may need to be adjusted or improved to improve the overall intervention effect.

6) The penultimate paragraph mentions "The researcher who delivered the intervention also conducted the post-intervention interviews, thus findings may be influenced by positive demand characteristics, particularly those relating to the delivery of the intervention.",and it is suggested to add relevant information in the method and result section, or explain why it is not mentioned.

7 Conclusion

Although the conclusion summarizes the main findings of the study and points out the potential of multi-component intervention strategy in reducing sedentary behavior of the elderly, there is still room for improvement. Conclusion There is a lack of discussion on how

the research results can guide the optimization of future intervention strategies, and the overall description is too general. It is suggested that the author further refine the conclusion and clearly explain the practical value of the research findings to health care providers and decision makers, so as to enhance the application significance and influence of the research. By providing more specific and operational insights, the conclusion will better guide practice and promote the development of this field.

**Reviewer**	**2**
**Name**	**Aubert, Carole**
**Affiliation**	**Inselspital University Hospital Bern, General Internal Medicine**
**Date**	**19-Jan-2025**
**COI**	**None**

This study assessed acceptability of an intervention to reduce sedentary behaviour in older adults. The topic is of importance. I suggest some modifications to iimprove the manuscript.

General comments

1) I suggest avoid unusual and multiple abbreviations, such as OA, SB, PA, NC, MVPA, IDI, REC, ...
2) There is a mix of US and UK English, please proof-read.

Abstract

1) Abstract: please clarify the goal of the interviews.
2) Abstract: the conclusion does not reflect the study methods. This article reports on a process evaluation of the intervention, and not on the results of the intervention. The conclusion saying that the intervention has the potential to reduce sedentary behaviour is thus not appropriate.
3) The study title and the strengths underscore the “multi-ethnic” population, but there is nothing about that in the abstract. I suggest either to add to the abstract or to change the title / strength section.

Introduction

1) I suggest clarifying what are the negative impacts of sedentary behaviour specifically in older adults.
2) Page 2, line 22: what does “2020a” mean?
3) The introduction is very long and not well focusing. I suggest some rework and shortening.
4) The authors underscore how important it is to assess acceptability of the intervention. While this is correct, it is also (maybe even more important) to co-design interventions with end-users instead of collecting this information only on the developed intervention. Please comment on this issue.
5) Page 4, lines 51-52: high acceptability and satisfaction about an intervention does not allow to conclude that the intervention can effectively reduce sedentary behaviour. Please correct.
6) The authors refer alternatively to “multi-ethnic” and “ethnic minorities”, which is not the same. Please clarify and be more consistent.
7) The title suggests that the study assesses acceptability of a specific intervention (12-week program), but in the last paragraph of the introduction, the authors mention that their study will assess “the effects of interventions”, suggesting multiple interventions. Furthermore, this study does not assess the effects, but rather does a process evaluation of the intervention. The last sentence of this last paragraph is more correct.

Methods

1) The lack of patient and public involvement in such a study is a big limitation. Please comment.
2) Please comment on recruitment bias in the recruitment methods.
3) I suggest to better describe the types of populations / ethnics that were attending the settings where recruitment was done.
4) Please describe briefly the 12-week intervention.
5) Please comment on sample size calculation.
6) Data analysis: please clarify what is meant by “the transcripts were returned”.
7) The interview guide has a lot of close-ended questions, which is little appropriate for qualitative research. Please comment.

Results

1) The authors report almost only positive feedback, except for the WAT. Even if it is possible that it was so well welcome, it is weird that it was received almost only positively or without more suggestions for adaptation / improvement. Has there been any selection of quotes that is uneven? The interview guide indeed still has few questions asking for suggestions for improvement. Please comment / clarify.
2) Some close-ended questions of the interviews seem to try to quantify something (e.g., about the duration of the intervention) and the answers are also reported as such in the results. This would be rather quantitative analysis and the sample size is questionable for such analysis. Please comment.
3) The suggestion of adding incentives is questionable, since the goal should not be to participate in a study, but to receive an intervention that help older adults improve their health. Please comment.
4) Page 15, paragraph 2 (level of interest): I don’t understand the sentence marking a contradiction (with “despite”). Moving more is indeed related to staying fit, and not opposed to it. Please clarify.
5) Role of family: the first sentence seems to have something wrong grammatically. Please correct.

Discussion

1) The discussion is very long and all paragraphs could be shorter and more focused. I suggest some rework.
2) Page 18, second paragraph: it would be more relevant to focus on how to retain older adults in a real-life program than in a study.
3) Page 18, third paragraph: similarly, the authors underscore the importance of incentives / financial compensation for participating. Please comment on how this would work in real-life, while the goal is finally not just to be able to do an intervention in research conditions, but have it implemented in real life. While the burden of questionnaires / IC signature could be compensated, this should not be the case for an intervention, for which participants might even have to pay in real life! Please comment.
4) Page 19, paragraph 2: one more paragraph really focused on research. This makes a lot of the discussion focused on research and not on real-life implementation.
5) Conclusion: same comment as for the conclusion of the abstract.

Tables and figures

1) Table 1: Gender refers to men/women, while sex refers to male/female. Please correct as appropriate.
2) Table 2: please clarify why some subthemes are in italics and others not.
3) Figure: what does that add compared to Table 2? It is the same, but in a figure instead of a table.

VERSION 1 - AUTHOR RESPONSE

**Comments to Author:**	**Responses**:
**General comments**<br><br>1) I suggest avoiding unusual and multiple abbreviations, such as OA, SB, PA, NC, MVPA, IDI, REC, ...<br><br>2) There is a mix of US and UK English, please proof-read	Thanks for your feedback.<br>We have avoided unnecessary abbreviations and proof-read English throughout the document.
**Abstract**<br>1) Abstract: please clarify the goal of the interviews.<br>2) Abstract: the conclusion does not reflect the study methods. This article reports on a process<br>evaluation of the intervention, and not on the results of the intervention. The conclusion<br>saying that the intervention has the potential to reduce sedentary behaviour is thus not<br>appropriate.<br>3) The study title and the strengths underscore the “multi-ethnic” population, but there is nothing<br>about that in the abstract. I suggest either to add to the abstract or to change the title / strength<br>section.	Thanks for your feedback. We have revised the entire abstract. Changes are done on page 1.
**Introduction**<br>1) I suggest clarifying what are the negative impacts of sedentary behaviour specifically in older<br>adults.	Thanks for your feedback. We have added the negative impacts of sedentary behaviour. Changes are done on page 3, line no. 14 – 35.
2) Page 2, line 22: what does “2020a” mean?	Thanks for highlighting it we have removed this typo error.
3) The introduction is very long and not well focusing. I suggest some rework and shortening.	Thanks for your feedback. We have removed unnecessary details and made relevant changes. Changes are highlighted on page 4, line no. 9 -28.
4) The authors underscore how important it is to assess acceptability of the intervention. While<br>this is correct, it is also (maybe even more important) to co-design interventions with end users instead of collecting this information only on the developed intervention. Please comment on this issue.	Thanks for your feedback. We have added relevant details.<br><br>Changes are highlighted on page 4, line no. 21 -28.

5) Page 4, lines 51-52: high acceptability and satisfaction about an intervention does not allow to<br>conclude that the intervention can effectively reduce sedentary behaviour. Please correct.	Thanks for your feedback. We have added relevant details.<br><br>Changes are highlighted on page 4, line no. 21 -28.
6) The authors refer alternatively to "multi-ethnic" and "ethnic minorities", which is not the same. Please clarify and be more consistent.	Thanks for your feedback. We have consistently used ethnically diverse older adults.
7) The title suggests that the study assesses acceptability of a specific intervention (12-week<br>program), but in the last paragraph of the introduction, the authors mention that their study<br>will assess "the effects of interventions", suggesting multiple interventions. Furthermore, this<br>study does not assess the effects, but rather does a process evaluation of the intervention. The<br>last sentence of this last paragraph is more correct	Thanks for your feedback. We have corrected the suggested error.
**Methods**<br>1) The lack of patient and public involvement in such a study is a big limitation. Please comment.	Thanks for your feedback. As commented in the Introduction, through an in-depth analysis of data obtained from older adults, we gained important knowledge and built insight into the complexity of physical activity and sedentary behaviour in this group. These findings provide a suitable underpinning to design and execute a multi-component person-centred intervention for community dwelling ethnically diverse OAs which was examined for acceptability in the current study. However, in a future effectiveness trial, the PPI principle can be followed to integrate end users and stakeholders throughout the study.
2) Please comment on recruitment bias in the recruitment methods.	Thanks for your feedback. We admit that there may have been some recruitment bias introduced by the recruitment procedure, which involved local religious organisations and social community groups that provided recreational activities. This strategy can encourage the inclusion of older adults who are already involved in sports and leisure activities, or socially active. Therefore, we also included older adults who were currently not affiliated with these organizations but were previously part of these groups. However, in future, adding more recruitment channels, like healthcare facilities, social service organisations, or larger public

	outreach campaigns can reduce this potential bias and include a wider variety of older adults.
3) I suggest to better describe the types of populations / ethnics that were attending the settings where recruitment was done.	Thanks for your feedback. We have added types of population. Changes are highlighted on page 5, line no. 14- 16.
4) Please describe briefly the 12-week intervention.	Thanks for your feedback. We have added brief details and also provided reference for the protocol paper for further details. Changes are done on page 5, line no. 29-33
5) Please comment on sample size calculation.	Thanks for your feedback. We have added details regarding sample size. Changes are done on page 5, line no. 9 – 12.
6) Data analysis: please clarify what is meant by "the transcripts were returned".	Thanks for your feedback. The sentence has been modified. Changes are done on page 6 line no.38
7) The interview guide has a lot of close-ended questions, which is little appropriate for qualitative research. Please	Thanks for your feedback. We have added brief response:<br>Though some questions were closed-ended in the interview guide, these were included to introduce topics within the conversation and the addition of probes helped the researcher to explore in-depth insights from participants' responses.
**Results**<br>1) The authors report almost only positive feedback, except for the WAT. Even if it is possible that it was so well welcome, it is weird that it was received almost only positively or without more suggestions for adaptation / improvement. Has there been any selection of quotes that is uneven? The interview guide indeed still has few questions asking for suggestions for improvement. Please comment / clarify.	Thanks for your feedback. We also included some of the feedback from the participants. Changes are done on page 9 line 27-30 and on page 10, line 10-14
2) Some close-ended questions of the interviews seem to try to quantify something (e.g., about the duration of the intervention) and the answers are also reported as such in the results. This would be rather quantitative analysis and the sample size is questionable for such analysis. Please comment.	Thanks for sharing your thoughtful comments. Although there were some closed-ended interview questions, our main goal was to explore participant perspectives rather than perform a quantitative study. By emphasising frequent trends or preferences for the duration of the intervention, these responses served to enhance and support the qualitative findings which help us to report how participants perceive the intervention's duration.
3) The suggestion of adding incentives is questionable, since the goal should not be to participate in a study, but to receive an intervention that help older adults improve their health. Please comment.	Thanks for your comment. We recognise that the use of incentives raises concerns. Some older adults who participated directly suggested adding incentives, believing that a small incentive could recognise the time and effort needed to participate in the intervention research, especially in a demographic

	that might have time constraints. We do agree, though, that the intervention's possible health benefits should serve as the main driving force. If incentives are taken into account, they would be planned to reduce excessive influence and would follow ethical standards, making sure they don't take precedence over the study's health-focused objectives.
4) Page 15, paragraph 2 (level of interest): I don't understand the sentence marking a contradiction (with "despite"). Moving more is indeed related to staying fit, and not opposed to it. Please clarify.	Thanks for your feedback. We modified sentence. Changes are done on page 14, line 17
5) Role of family: the first sentence seems to have something wrong grammatically. Please correct.	Thanks for your feedback. We modified sentence. Changes are done on page 9 line 27-30 and on page 14, line 28
The discussion is very long and all paragraphs could be shorter and more focused. I suggest some rework.	Thanks for your feedback. We have removed irrelevant changes.
2) Page 18, second paragraph: it would be more relevant to focus on how to retain older adults in a real-life program than in a study	Thanks for your feedback. Changes are done on page 16, line 29-38 & pg 17 1-10
3) Page 18, third paragraph: similarly, the authors underscore the importance of incentives / financial compensation for participating. Please comment on how this would work in real-life, while the goal is finally not just to be able to do an intervention in research conditions, but have it implemented in real life. While the burden of questionnaires / IC signature could be compensated, this should not be the case for an intervention, for which participants might even have to pay in real life! Please comment.	Thanks for your feedback. Changes are done on page 17, line 12-24
4) Page 19, paragraph 2: one more paragraph really focused on research. This makes a lot of the discussion focused on research and not on real-life implementation.	Thanks for your feedback. Changes are done on page 17, line 25-38 & page 18 line 16-29
5) Conclusion: same comment as for the conclusion of the abstract.	Thanks for your feedback. Changes are done on page 18, line 6 -14
Tables and figures 1) Table 1: Gender refers to men/women, while sex refers to male/female. Please correct as appropriate	Thanks for your feedback. Changes are done on page 21
2) Table 2: please clarify why some subthemes are in italics and others not.	Thanks for your feedback. This typo error has been corrected

. 3) Figure: what does that add compared to Table 2? It is the same, but in a figure instead of a table	Thanks for your feedback. Figure has been removed

---

VERSION 2 - AUTHOR RESPONSE

Reviewer: 1<br>Miss Yufang Hao, Beijing University of Chinese Medicine<br>Comments to the Author:<br>This manuscript is a study of great public health significance, focusing on the improvement of health behavior of multi-ethnic elderly groups. The research design is reasonable and the intervention measures are clear, which can effectively evaluate the behavior changes of the target population. The advantage of the manuscript lies in its concern for multi-ethnic groups, which helps to reveal the influence of cultural factors on the acceptability of intervention. However, the manuscript still needs to be improved in some aspects, especially in the preface, the clear definition of acceptability and sedentary, the detailed description of methods, the specific analysis of results, and the logic and depth of discussion.	Thanks for your feedback. We have done necessary changes.
1 Title<br>1) Punctuation should not be added at the end of the title, and it is suggested to delete it.<br>2) The intervention strategy in this paper is designed to reduce sedentary behavior. In fact, the intervention measures to reduce sedentary behavior may not necessarily increase physical activity (similarly, the intervention measures to increase physical activity may not necessarily reduce sedentary behavior). Therefore, the title mentions reduce sedentary behavior and increase physical activity, which needs to be considered again.	Thanks for your feedback.<br>We have revised the title. Changes are done on page 1, line no. 2-3.
2 Abstract<br>1) Introduction: It is suggested to emphasize the importance of sedentary behavior and physical activity in multi-ethnic elderly groups, and point out the research gaps in this field to highlight the	We have revised the title. Changes are done on page 2, line no. 1-30.

necessity of research.<br>2) Methods: It is suggested to adjust the expression of "Underpinned by the themes discussed below", which can be directly defined as "four pre-determined MRC framework themes" to improve the clarity of expression. Also, it is suggested that the number of participants be clearly stated.<br>3) Conclusion: The conclusion should be consistent with the purpose of the study and emphasize the influence of the study on the multi-ethnic elderly. Moreover, the abbreviation "SB" should be marked with its full name for the first time	
3 Introduction<br>1) The preface mentions the concepts of sedentary behavior and physical activity, but it lacks a clear distinction. It is suggested to add a paragraph to explain the difference between the two concepts of "sedentary behavior" and "insufficient physical activity" in order to avoid confusion among readers.	Thanks for your feedback. We have incorporated your feedback. Changes are done on page 4, line no. 22- 33.
2) Please define the specific time of sedentary behavior in this study, which is the cumulative daily time or the single sitting time?	Thanks for your feedback. We have incorporated your feedback. Changes are done on page 5, line no. 8-9.
3) Although the author mentioned the importance of "acceptability", he did not clearly define this concept and did not fully elaborate its significance. This is an important aspect that needs to be improved. Please add more explanations and provide relevant quotations.	Thanks for your feedback. We have incorporated your feedback. Changes are done on page 5, line no. 30-38 and page 6, line no. 1-25.
4) The first paragraph mentioned "There has been an increase in concern about these findings following the imposition of COVID-19 constraints during the pandemic, as sedentary behaviour (SB) has become more pronounced and physical activity (PA) levels have lessened compared to pre pandemic levels (8).", but this study was actually carried out after the epidemic, so it is suggested to supplement the latest data or research on the changes of physical activity level after the epidemic to provide more complete background information.	Thanks for your feedback. We have incorporated your feedback. Changes are done on page 4, line no. 30-38 and page 6, line no. 1-25.
5) It is suggested that it is necessary to further elaborate the intervention research on sedentary behavior and physical activity	Thanks for your feedback. We have incorporated your feedback. Changes are done on page 4, line no. 8-24 and page 5, line no. 13-22.

in multi-ethnic elderly groups. For example, supplement the data related to sedentary epidemic of ethnic minority elderly people, emphasize how the specific health challenges and cultural differences of these groups affect their health behaviors, and how the current research can fill the gaps in this field.	
6) Page 4, lines 18-22, lines 51-53, etc., please provide necessary references.	Thanks for your feedback. References have been added.
7) The appearance of MVPA in introduction does not specify what it means.	Thanks for your feedback. We have incorporated your feedback. Changes are done on page 4, line no. 27.
4 Methods<br>1) It is suggested that the author supplement the research design flow chart to show the research process intuitively.	Thanks for your feedback. Changes have been done on page 7-line no. 23
2) The ethical approval number is missing in the study design, please add it.	Thanks for your feedback. Changes have been done on page 6-line no. 37-38.
3) Rationality of including the standard: in the part of "self-reported sedentary time ≥5 hours a day", the author needs to explain the basis for choosing this standard (whether it is based on previous research or guidelines) and its relevance to acceptability research.	Thanks for your point; it is really interesting. However, as there is no international agreed definition of the “amount” of sedentary time to identify an individual as sedentary, we will just report sedentary time and will not identify the participants as a sedentary group. As the Ekelund harmonised meta-analysis (1) illustrates, there are significant increased risks with increasing ST on all-cause mortality which is how the 5h threshold was derived. In addition, since we have used self-reported sitting time, this is likely an under-estimation, as evident in literature (2)<br>1. Ekelund U, Steene-Johannessen J, Brown WJ, Fagerland MW, Owen N, Powell KE, et al. Does physical activity attenuate, or even eliminate, the detrimental association of sitting time with mortality? A harmonised meta-analysis of data from more than 1 million men and women. The lancet. 2016;388(10051):1302-10.<br>2. Dohrn I-M, Gardiner PA, Winkler E, Welmer A-K. Device-measured sedentary behavior and physical activity in older adults differ by demographic and health-related factors. European Review of Aging and Physical Activity. 2020;17:1-11.
4) In the section of "Patient and public involvement", please describe the patient and public participation in the whole research process, not just setting the research agenda. If there is no PPI, it is suggested to explain why there is no PPI	Thanks for your feedback. As commented in the Introduction, through an in-depth analysis of data obtained from older adults, we gained important knowledge and built insight into the complexity of physical activity and sedentary behaviour in this group. These findings provide a suitable

and how this may affect the research.	underpinning to design and execute a multi-component person-centred intervention for community dwelling ethnically diverse OAs which was examined for acceptability in the current study. However, in a future effectiveness trial, the PPI principle can be followed to integrate end users and stakeholders throughout the study.
5) In the section of "Data Analysis", it is said that "four pre-determined MRC framework these are focused on the study's aims were employed", and it is suggested to clearly explain how to determine these four topics from the MRC framework and explain their relevance to the research objectives.	Thanks for your feedback. We have incorporated your feedback. Changes are done on page 8, line no. 32-36.
6) Please supplement the process or standard for determining the sample size. The proposal explains how to determine that data saturation has been achieved, especially considering the different views that may be brought by multi-ethnic participants.	Thanks for your feedback. We have incorporated your feedback. Changes are done on page 7, line no. 10-14.<br><br>In addition, to be clear, the acceptability interviews were conducted with all participants who participated in the feasibility intervention. This method made sure that the entire spectrum of participant experiences—including any differences in viewpoints among various ethnic backgrounds—was recorded.<br><br>Since each participant of the feasibility cohort was eligible to take part in the interviews, we systematically examined the responses to ascertain that data saturation was reached when no new themes appeared across multiple interviews. This was carried out while taking into account the participants' diversity, ensuring that the analysis included significant opinions from various ethnic groups.
7) Research intervention: Although the author mentioned that intervention is based on habit forming model design, he did not clearly describe how to form model design based on habit, and suggested adding it to increase clarity. At the same time, it is suggested to provide the details of the research intervention in the form of attachments, including the specific use methods, examples and implementation details of each component of the intervention	Thank you for your response. Based on your feedback, we have cited the protocol paper, which clearly describes the design based on habit. In addition, a detailed description of each intervention component is also included in the protocol paper. The intervention material has been attached as supplementary file

5 Results<br>1) The naming and division of topics and sub-topics need further discussion: a. It is suggested to adjust the naming of sub-topics under "Theme 1: Acceptability" to make them consistent in bias. For example, change "Effective and Simple" to neutral description. B the sub-topic "Convenience" under "theme 1: acceptability" overlaps with some other sub-topics, so it is suggested to re-examine and integrate the content; C. The sub-theme "Habit automation and conscious habit" under theme 3 suggests that "Habit Automation" and "Conscious Habit" should be merged or further condensed to improve the clarity; D for "Mediators" in theme 4, it is suggested to clarify whether its definition and use are appropriate.	Thanks for your response. We have included theme Acceptability based on the MRC framework. The sub-theme Convenience has been merged. Changes are done on page 11, line no. 11-21. The sub-theme “Habit automation and conscious habit" under theme 3 has been merged. Changes are done on page 10-line no. 9-28. The sub theme “Mediators" is merged. Changes are done on page 10, line no. 19-29. Effective and simple has been changed into Practicality of the Intervention. Changes are done on page 9, line no. 37.
2) The results lack the specific results of multi-ethnic groups, so it is suggested to report the special performance of multi-ethnic groups in the results and discuss the influence of cultural factors on the acceptability of intervention.	Thank you for your valuable feedback. This study predominantly focused on a multi-ethnic population, with the exception of two British participants. This makes our research unique compared to previous studies, as all perceptions and quotations presented in the results are directly related to the acceptability of the intervention within diverse cultural contexts. Given this focus, the findings inherently reflect the influence of cultural factors on intervention acceptability. The finding of this study captures a broader understanding of how participants from different cultural backgrounds perceived and engaged with the intervention.
3) It is suggested to describe the feedback of subjects to different components of multi-component intervention in order to better understand the effectiveness and acceptance of each component.	Thank you for your valuable feedback. Changes are done and highlighted in theme 2 Usability.
4) It is suggested that the author explain the quality of qualitative research, how to transfer, reliability, confirmability and credibility.	Thank you for your valuable feedback. Changes are done on page 9, line no. 3-14.
5) TABLE 1 does not provide the time for study participants to sit every day, please add it.	Thank you for your valuable feedback. Table has been updated. Changes are done on page 25.
6 Discussion<br>1) The third paragraph of the discussion lacks clear argument logic. The paragraph begins to discuss the importance of cognitive improvement, then jumps to habit formation, then to the role of	Thanks for your feedback. Entire discussion has been reworded. Changes are done and highlighted on page 17-20

reminding, and finally returns to the duration of intervention. This leap-forward discussion makes the central argument unclear, and it is suggested to reorganize the paragraph structure. In addition, this part mentioned the theory of habit formation, but did not fully explore how this theory specifically explained the research findings. The comparison with the existing literature is also general, lacking specific research citations and in-depth comparison.	
2) Similarly, in the third paragraph of the discussion, it is mentioned that reminding helps to automate behavior, but at the same time it is said that continuous reminding is needed. The relationship between these two viewpoints needs to be explained more clearly, because they actually describe different stages of behavior change and habit formation. In addition, it is mentioned that "with the passage of time, these may no longer be needed, because standing has become more automatic and habitual." "This statement is vague and fails to explain how long it will take to form a new habit. It is difficult to set intermediate checkpoints or evaluate progress standards for follow-up studies that lack time indicators.	Thanks for your feedback. Entire discussion has been reworded. Changes are done and highlighted on page 17-20
3) "This is aligned with the previous qualitative findings that identified OAs were interested in using technology as long as it is user-friendly."is mentioned in the first paragraph on page 17, please supplement the relevant references.	Thanks for your feedback. Changes are done and highlighted on page 18, line no. 10-14.
4) Consistent with the suggestions made in the results section, it is suggested to discuss the specific results of multi-ethnic groups in depth and analyze the significance and challenges of these characteristics in the intervention, especially how cultural factors affect the acceptability of the intervention.	Thanks for your feedback. Entire discussion has been reworded. Changes are done and highlighted on page 17-20
5) Consistent with the suggestions in the results section, it is suggested to discuss the acceptability of different components of multi-component intervention in detail, and point out which components may need	Thanks for your feedback. Entire discussion has been reworded. Changes are done and highlighted on page 17-20

to be adjusted or improved to improve the overall intervention effect.	
6) The penultimate paragraph mentions "The researcher who delivered the intervention also conducted the post-intervention interviews, thus findings may be influenced by positive demand characteristics, particularly those relating to the delivery of the intervention.",and it is suggested to add relevant information in the method and result section, or explain why it is not mentioned.	Thanks for your feedback. Changes are done and highlighted on page 6, line no. 33-34.
7 Conclusion<br>Although the conclusion summarizes the main findings of the study and points out the potential of multi-component intervention strategy in reducing sedentary behavior of the elderly, there is still room for improvement. Conclusion There is a lack of discussion on how the research results can guide the optimization of future intervention strategies, and the overall description is too general. It is suggested that the author further refine the conclusion and clearly explain the practical value of the research findings to health care providers and decision makers, so as to enhance the application significance and influence of the research. By providing more specific and operational insights, the conclusion will better guide practice and promote the development of this field.	Thanks for your feedback. Entire conclusion has been reworded. Changes are done page 20, line no. 36-38 and page 21, line no. 1-12.

VERSION 3 - AUTHOR RESPONSE

**Reviewer comment**	
We note a response to the following comment has not been provided. Please ensure the following reviewer comment is addressed:<br><br>2 Abstract<br>1) Introduction: It is suggested to emphasize the<br>importance of sedentary behavior and physical<br>activity in multi-ethnic elderly groups, and point out	Thanks for your feedback.<br>We have revised the abstract. Changes are done on page 2, line no. 1-38 and page 3, line no. 1-2.

the research gaps in this field to highlight the<br>necessity of research.<br>2) Methods: It is suggested to adjust the expression<br>of "Underpinned by the themes discussed below",<br>which can be directly defined as "four predetermined MRC framework themes" to improve the<br>clarity of expression. Also, it is suggested that the<br>number of participants be clearly stated.<br>3) Conclusion: The conclusion should be consistent<br>with the purpose of the study and emphasize the<br>influence of the study on the multi-ethnic elderly.	
- Please clarify what references were added in response to the following comment:<br>Reviewer comment:<br>6) Page 4, lines 18-22, lines 51-53, etc., please<br>provide necessary references.<br>Authors response:<br>Thanks for your feedback. References have been added.	Thanks for your feedback. We have added relevant details.<br><br>Changes are highlighted on page 6, line no 1.<br><br>36. Madhav K, Sherchand SP, Sherchan S. Association between screen time and depression among US adults. *Preventive medicine reports* 2017;8:67-71.<br>37. Colley RC, Garriguet D, Janssen I, et al. Physical activity of Canadian adults: accelerometer results from the 2007 to 2009 Canadian Health Measures Survey. *Health reports* 2011;22(1):7.<br><br>Changes are highlighted on page 6, line no 14-15.<br><br>50. Kerr J, Rosenberg D, Frank L. The role of the built environment in healthy aging: Community design, physical activity, and health among older adults. *Journal of planning Literature* 2012;27(1):43-60.<br>51. Cunningham GO, Michael YL. Concepts guiding the study of the impact of the built environment on physical activity for older adults: a review of the literature. *American journal of health promotion* 2004;18(6):435-43.<br>52. Michael YL, Green MK, Farquhar SA. Neighborhood design and active aging. *Health & place* 2006;12(4):734-40.

	doi: https://doi.org/10.1016%2Fj.healthplace.2005.08.002<br>53. Yen IH, Fandel Flood J, Thompson H, et al. How design of places promotes or inhibits mobility of older adults: realist synthesis of 20 years of research. *Journal of aging and health* 2014;26(8):1340-72. doi: https://doi.org/10.1177%2F0898264314527610<br>54. Won J, Lee C, Forjuoh SN, et al. Neighborhood safety factors associated with older adults' health-related outcomes: a systematic literature review. *Social Science & Medicine* 2016;165:177-86. doi: 10.1016/j.socscimed.2016.07.024<br>55. Levasseur M, Généreux M, Bruneau J-F, et al. Importance of proximity to resources, social support, transportation and neighborhood security for mobility and social participation in older adults: results from a scoping study. *BMC public health* 2015;15(1):1-19. doi: https://doi.org/10.1186/s12889-015-1824-0<br>56. Moran M, Van Cauwenberg J, Hercky-Linnewiel R, et al. Understanding the relationships between the physical environment and physical activity in older adults: a systematic review of qualitative studies. *International journal of behavioral nutrition and physical activity* 2014;11:1-12.<br>57. Matz CJ, Stieb DM, Davis K, et al. Effects of age, season, gender and urban-rural status on time-activity: Canadian Human Activity Pattern Survey 2 (CHAPS 2). *International journal of environmental research and public health* 2014;11(2):2108-24. doi: https://doi.org/10.3390/ijerph110202108
Reviewer comment:<br>2) The results lack the specific results of multi-ethnic groups, so it is suggested to report the special performance of multi-ethnic groups in the results and discuss the influence of cultural factors on the acceptability of intervention.<br>Authors Response:<br>Thank you for your valuable feedback. This study predominantly focused on a multi-ethnic population, with the exception of two British participants. This makes our research unique compared to previous	Thanks for your feedback. We have added relevant details.<br><br>Results:<br>Changes are done on pg 12, line no. 6-8.<br>Changes are done on pg 12, line no. 14-17.<br>Changes are done on pg 14, line no. 12-18.<br>Changes are done on pg 17, line no. 26-32.<br><br>Discussion:<br><br>Changes are done on pg. 19, line no. 37-38.<br>Changes are done on pg. 20, line no. 10-13.<br>Changes are done on pg. 20, line no. 20-22.<br>Changes are done on pg. 21, line no. 2-9

studies, as all perceptions and quotations presented in the results are directly related to the acceptability of the intervention within diverse cultural contexts. Given this focus, the findings inherently reflect the influence of cultural factors on intervention acceptability. The finding of this study captures a broader understanding of how participants from different cultural backgrounds perceived and engaged with the intervention.	
